# Highlighting the Potential of Synergistic Cu–Pt Single-Atom Alloy Sub-nanoclusters for Enhanced H_2_ Adsorption: A DFT Investigation

**DOI:** 10.1021/acsnanoscienceau.4c00058

**Published:** 2024-12-16

**Authors:** João Paulo Cerqueira Felix, Wanderson Souza Araújo, João Marcos Tomaz Palheta, Jônatas Favotto Dalmedico, Fabiano Pereira de Oliveira, Alexandre C. Dias, Diego Guedes-Sobrinho, Celso R. C. Rêgo, Renato L. T. Parreira, Maurício J. Piotrowski

**Affiliations:** † Institute of Physics “Armando Dias Tavares”, Rio de Janeiro State University, 20550-900 Rio de Janeiro, Rio de Janeiro, Brazil; ‡ Department of Physics, 37902 Federal University of Pelotas, PO Box 354, 96010-900 Pelotas, Rio Grande do Sul, Brazil; ¶ Institute of Physics and International Center of Physics, University of Brasília, 70919-970 Brasília, Federal District, Brazil; § Chemistry Department, Federal University of Paraná, 81531-980 Curitiba, Paraná, Brazil; ∥ Institute of Nanotechnology Hermann-von-Helmholtz-Platz, Karlsruhe Institute of Technology, 76021 Karlsruhe, Germany; ⊥ Núcleo de Pesquisas em Ciências Exatas e Tecnológicas, 92917 Universidade de Franca, 14404−600 Franca, São Paulo, Brazil

**Keywords:** Density functional theory, Cu and CuPt sub-nanoclusters, H_2_ molecule, molecular adsorption, single-atom alloy

## Abstract

Single-atom alloy sub-nanoclusters offer promising potential for understanding intricate interfacial phenomena at the atomic level, enabling the rational design of efficient catalysts and nanomaterials for H_2_ energy storage, purification, and conversion. Herein, we employed density functional theory calculations improved by van der Waals corrections to investigate H_2_ adsorption on pure copper (Cu_*n*_) and copper–platinum (Cu_*n*–1_Pt) sub-nanoclusters. We characterized Cu_*n*_ sub-nanoclusters ranging from *n* = 2 to *n* = 14, identifying the most stable sizes (4, 6, 8, 10, and 12) through a set of stability analysis. Subsequently, we substituted a single Cu atom with Pt to form single-atom alloy Cu_*n*–1_Pt sub-nanoclusters, which showed enhanced stabilization and reactivity compared to pure Cu sub-nanoclusters. While Cu-only sub-nanoclusters exhibited weak side-on interactions with H_2_, resulting in minimal charge transfer and negligible structural changes, CuPt-based sub-nanoclusters showed strong interactions characterized by molecular dissociation (H–H bond breaking) and significant charge transfer from the sub-nanoclusters to the H atoms. These findings highlight the synergistic effects of the Cu–Pt combination and provide valuable insights into the fundamental processes of H_2_ adsorption on metal sub-nanoclusters, with significant implications for catalytic applications and materials design in hydrogen-related technologies.

## Graphical abstract

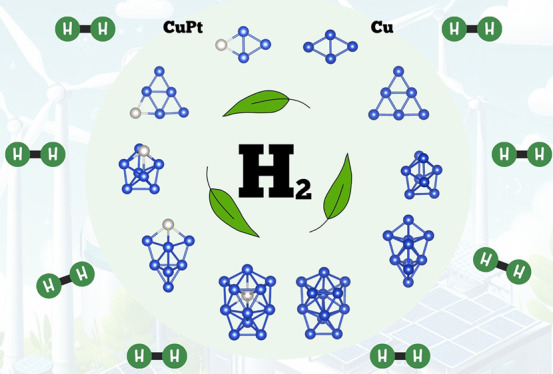

## 1 Introduction

Nanocluster metal-containing catalysts are essential in heterogeneous, homogeneous, and enzymatic catalysis, playing a pivotal role in achieving high selectivity, thermal stability, recyclability, and effective product-catalyst separation. These features are crucial in many fields, especially in the quest for sustainable energy generation.^1–3^ Among the promising candidates for efficient catalysts are metallic sub-nanoclusters, which are composed of a few atoms and exhibit unique physical and chemical properties.^4–6^ In the realm of energy generation and storage, hydrogen (H_2_) stands out for its potential to store energy through chemical bonds.^7^ The current landscape highlights a stark contrast between the production of H_2_ from renewable sources, known as green hydrogen, and fossil fuels.^8^

Green hydrogen production methods, such as water electrolysis, catalysis, and biomass conversion, aim to minimize environmental impact by generating hydrogen without residual pollution. Thus, experimental and theoretical studies of metallic nanoclusters and, more recently, single-atom alloy (SAA) nanoclusters^9^ have identified them as promising catalysts.^3,6,10,11^ SAAs are a type of bimetallic catalyst where isolated atoms of one metal (often a noble or transition metal) are dispersed within the host lattice of another metal, forming a dilute alloy. In these systems, the single atoms serve as the active sites for catalytic reactions, while the surrounding metal atoms influence these sites’ electronic properties and stability. The unique combination of atomic dispersion and the synergistic effects between the different metals in SAAs often leads to enhanced catalytic performance, including improved selectivity and activity compared to traditional bimetallic catalysts.

Catalysis, mainly via water electrolysis, is crucial for green hydrogen production. However, a significant barrier persists in the sluggish kinetics of water reduction to H_2_ and oxidation to O_2_.^12^ Overcoming this challenge requires the discovery of efficient and durable catalysts. Metallic sub-nanoclusters have garnered significant attention due to their diverse applications in catalysis.^10,13,14^ These sub-nanoclusters exhibit enhanced reactivity and catalytic efficiency compared to bulk materials.^4,5^ SAA systems enhance these properties by atomically dispersing one metal throughout the catalyst via alloy bonding. This approach combines the advantages of alloy catalysts with the unique properties of single-atom catalysts (SAC).^15^ The peculiar electronic structures of SACs can be finely tuned by adjacent bonding atoms through strong metal–support interaction and/or confinement.

Considerable research has focused on understanding the relationship between structure and reactivity in sub-nanoclusters, SACs, and SAAs to improve catalytic performance; however, the complexity of these systems has made their rational design challenging, and they remain an area of intense research.^2^ SAAs, in particular, consist of single atoms of a catalytically active metal alloyed into the surface layer of a less reactive host metal. In the context of nanoclusters, this can be thought of as single atoms of metal A dispersed on nanoclusters of metal B.^16,17^ SAAs offer unique properties, including improved activity and selectivity compared to monometallic counterparts and resistance to deactivation and poisoning.^9,16^ Most research on these materials has focused on trace amounts of group 8–10 transition metals alloyed into group 11 metals.^16–18^

Therefore, among metallic nanoclusters, copper (Cu) sub-nanoclusters stand out for their exceptional catalytic activity, electrical conductivity, and biocompatibility, making them highly desirable for applications in catalysis, electronics, and biomedicine.^19,20^ Understanding the structural stability, electronic properties, and reactivity of Cu sub-nanoclusters is crucial for tailoring their functionalities for specific applications.^21^ Recent advancements in computational methods, particularly density functional theory (DFT), have facilitated accurate predictions of these properties, enabling the rational design and optimization of Cu sub-nanoclusters.^22,23^ In addition to standalone Cu sub-nanoclusters, there is growing interest in exploring the behavior of Pt-based SAAs, where individual Pt atoms are dispersed within a matrix of another metal.^24^ SAAs exhibit unique catalytic properties arising from synergistic interactions between the dispersed metal atoms and the Cu matrix, offering enhanced catalytic performance and selectivity compared to pure metal catalysts.^25^

Understanding the interaction between H_2_ and Cu/CuPt sub-nanoclusters is crucial for advancing green hydrogen production catalysis since hydrogen adsorption on metal nanoclusters is particularly relevant for hydrogen storage and purification processes, as well as hydrogenation reactions in catalysis.^19,20,26,27^ Insights from such studies are instrumental in engineering nanomaterials tailored for sustainable energy technologies. By elucidating the interaction mechanisms between H_2_ molecules and Cu and CuPt sub-nanoclusters, this research aims to provide insights into the fundamental processes governing H_2_ adsorption on metal nanosystems, which is essential for developing efficient hydrogen storage materials and catalysts.

Thus, this study investigates, via DFT calculations, the structural stability and electronic properties of Cu_*n*_ and Cu_*n*–1_ Pt sub-nanoclusters (*n* = 2 to 14), where a single Cu atom is substituted with a Pt atom. Incorporating Pt into Cu sub-nanoclusters enhance their catalytic performance and stability, making Cu_*n*–1_Pt sub-nanoclusters promising candidates for H_2_ adsorption and dissociation. By comprehensively characterizing the structural, electronic, and adsorption properties of H_2_ on Cu_*n*_ and Cu_*n*–1_Pt sub-nanoclusters, we aim to provide fundamental insights into their behavior at the nanoscale. The findings presented here can help design novel catalysts and materials with tailored functionalities for H_2_ applications.

## 2 Methodology

### 2.1 Computational Details

All calculations in this study were based on spin-polarized DFT^28,29^ by using the (semilocal) generalized gradient approximation for the exchange-correlation energy functional, as proposed by Perdew, Burke, and Ernzerhof (PBE).^30^ Empirical D3 corrections, as proposed by Grimme,^31,32^ were included to account for attractive nonlocal long-range van der Waals (vdW) interactions. The Kohn–Sham (KS) equations were solved using the all-electron projected augmented wave (PAW) method,^33,34^ as implemented in the Vienna *Ab initio* Simulation Package (VASP).^35,36^ In this approach, the valence electrons were treated using a scalar-relativistic approximation, while the core electrons were described by fully relativistic calculations.^37,38^

For the expansion of the KS orbitals, we considered plane waves up to a cutoff energy of 500 eV for all calculations, which is 20% higher than the highest value recommended by VASP (ENMAX) among the selected PAW files. For density of states (DOS) and total energy calculations, we used a cutoff energy value that was 1.5 times higher. Our nonperiodic systems were simulated in a cubic box with a side length of 20 Å, providing a minimum separation distance of approximately 12 Å, which is enough to avoid the self-interaction for the largest systems and their periodic images. For Brillouin zone integration, a single **k**-point (Γ-point) was used for the sub-nanoclusters, molecules, and free atoms. A small Gaussian smearing parameter of 1 meV was applied to prevent fractional occupation of electronic states.

The convergence criteria for all optimizations were set as follows: for the ionic loop, equilibrium geometries were considered achieved when the atomic forces on every atom were smaller than 0.015 eV/Å, and for the electronic loop, the total energy convergence criterion was 1.0 × 10^–6^ eV for electronic self-consistency. Additional details regarding the convergence tests are provided in the Supporting Information (SI) (Tables S1–S4). To determine the 3*N* – 6 (3*N* – 5) vibrational modes for three-dimensional (two-dimensional) systems, the Hessian matrix was calculated using finite differences, as implemented in VASP. This involved displacing each atom in each direction by ±0.01 Å. To confirm the thermodynamic stability of the most stable Cu and CuPt sub-nanoclusters, we employed *Ab Initio* Molecular Dynamics (AIMD) simulations using the Born–Oppenheimer dynamics approach.^39^ These simulations began with the optimized configurations and aimed to verify thermodynamic stability through a thermalization procedure at 300 K, employing a Nosé–Hoover thermostat within an NVT ensemble.^40,41^ The AIMD simulations were conducted over 5 ps with a time step of 1 fs, resulting in final configurations that were subsequently structurally optimized using DFT-PBE+D3 calculations.

### 2.2 Atomic Configurations

We constructed a set of representative structural motifs following the structural design principles outlined in our previous works.^23,42^ From this set, we obtained the putative global minimum configurations, or the lowest energy configurations, of Cu_*n*_ (*n* = 2–14) sub-nanometric clusters (clu), as shown in Figure 1(a). Our representative set’s structural and magnetic diversity was based on physical principles such as symmetry and dimensionality. These included linear, planar, globular, compact, open, highly symmetric, and amorphous geometries. We also tested different spin configurations (magnetic-like orderings) and performed structural crossovers among the putative global minima for each size and transition metal species, as detailed in the systematic study by Chaves et al.^42^ for all 30 transition metal elements. Consequently, the lowest energy configurations of the Cu sub-nanoclusters were obtained from DFT-PBE+D3 optimizations without imposing any geometric constraints. These results are in complete agreement with the literature,^23,42^ and the inclusion of the vdW D3 corrections did not alter the most stable configurations.^43,44^

**Figure 1:**
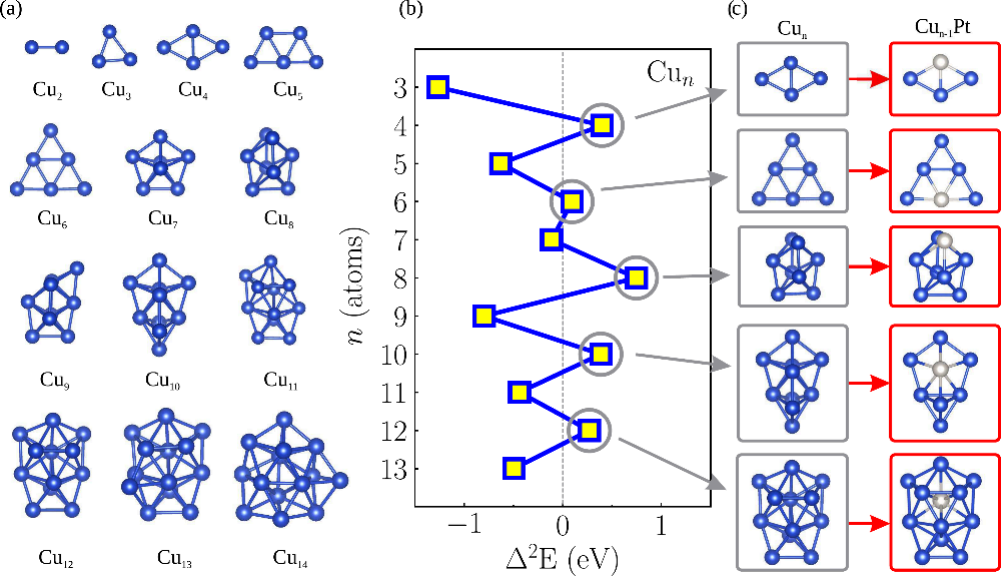
Methodological process for selecting sub-nanocluster substrates: (a) the lowest energy structures of Cu_*n*_ (*n* = 2–14) sub-nanoclusters; (b) the stability function Δ^2^ *E versus* the number of Cu atoms (*n*); (c) the most stable sizes for Cu_*n*_ sub-nanoclusters and the corresponding most stable Cu_*n*–1_Pt sub-nanoclusters.

For the H_2_ adsorption step, we selected certain sub-nanoclusters to serve as substrates. To identify the most stable sizes of the Cu_*n*_ sub-nanoclusters, we used the stability function, Δ^2^ *E*,^22,45^ a well-established energetic stability criterion in the context of sub-nanoclusters.^42,46,47^ The adapted equation for the stability function within the range of *n* = 2–14 is given by

1 $$\Delta^{2}E=E_{tot}^{{Cu}_{n-1}}+E_{tot}^{{Cu}_{n-1}}-2E_{tot}^{{Cu}_{n}}$$

where $E_{tot}^{{Cu}_{n}}$ represents the total energy of the Cu_*n*_ sub-nanoclusters. Figure 1(b) illustrates the evolution of Δ^2^ *E* for Cu_*n*_ sub-nanoclusters as a function of the number of Cu atoms. Despite the entropic effects in the experimental apparatus for cluster generation, which produces many isomers, the sizes with remarkable stability are the most probable structures.^48^

In addition to these configurations, we also constructed Cu_*n*–1_Pt-type structures, which can be classified as SAA catalysts.^9^ These sub-nanoclusters predominantly consist of Cu (acting as the selective host) with a single Pt atom (serving as the reactive dopant), designed to leverage the enhanced chemical activity of Pt in a single-atom context.^15^ Figure 1(c) displays the lowest energy sizes for Cu_*n*_ sub-nanoclusters and the corresponding most stable Cu_*n*–1_Pt sub-nanoclusters. These Cu_*n*–1_Pt configurations were identified through a comprehensive search for the most stable Pt site among all possible nonequivalent Cu sites in each sub-nanocluster size, followed by DFT-PBE+D3 optimization.

Using the two sets of configurations shown in Figure 1(c) as substrates, we explored the H_2_ molecular adsorption process, considering two bonding modes: H_2_ side-on and H_2_ end-on.^49^ We identified the lowest-energy adsorption sites by examining all nonequivalent positions. Following an approach similar to that used for low-Miller-index surfaces, we evaluated onefold (top), 2-fold (bridge), and 3-fold (hollow) sites on the sub-nanoclusters. However, the chemical diversity of the sub-nanoclusters increased the number of adsorption possibilities. This diversity stems from variations in coordination numbers and chemical compositions, particularly for Cu_*n*–1_Pt sub-nanoclusters in both H_2_-bonding modes. Consequently, we systematically considered all nonequivalent sites with distinct coordination and composition, including top, bridge, and hollow sites with varying chemical neighborhoods, placing the H_2_ molecule in both side-on and end-on orientations on the sub-nanoclusters.

### 2.3 Property Analysis

In addition to the stability function and the relative total energy, both crucial for selecting stable configurations and sizes, we conducted an analysis of energetic properties, including the binding energy per atom, *E*_b_^clu^:

2 $$E_{b}^{clu}=\frac{E_{tot}^{clu}-nE_{tot}^{Cu}-mE_{tot}^{Pt}}{n+m}$$

where *E*_tot_^clu^ represents the total energy calculated for the sub-nanoclusters, while *E*_tot_^Cu^, *E*_tot_^Pt^, *n* and *m* correspond to the total energies and number of atoms of the Cu and Pt atomic species, respectively, so that *m* = 0 for Cu_*n*_ and *m* = 1 for Cu_*n*–1_Pt. We also calculated Δ*E*_b_^clu^, which compares the variations of *E*_b_^clu^ when substituting a Cu atom with a Pt atom in the sub-nanoclusters:

3 $$\Delta E_{b}^{clu}=\frac{\left(E_{b}^{{Cu}_{n-1}Pt}-E_{b}^{{Cu}_{n}}\right)\times 100}{E_{b}^{{Cu}_{n}}}$$

This equation assesses the change in *E*_b_^clu^ as a percentage when transitioning from Cu to Pt atom incorporation in the sub-nanoclusters, providing insights into the impact of alloying on binding energies.

For the energetic analysis of H_2_/clu systems, we computed both binding and adsorption energies by considering the total energies of individual constituents and the interaction between H_2_ and the sub-nanocluster.^47,50,51^ The adsorption energy, *E*_ads_, is defined as

4 $$E_{ads}=E_{tot}^{H_{2}/clu}-E_{tot}^{clu}-E_{tot}^{H_{2}}=\Delta E_{\mathrm{int}}+\left(n+m\right)\Delta E_{dis}^{clu}+\Delta E_{dis}^{H_{2}}$$

where $E_{tot}^{H_{2}/clu}$ represents the total energy of the H_2_/clu system. Δ*E*_int_ is defined as

5 $$\Delta E_{\mathrm{int}}=E_{tot}^{H_{2}/clu}-E_{tot}^{clu\;frozen}-E_{tot}^{H_{2}frozen}$$

Distortion energies, Δ*E*_dis_^clu^ (per atom) and $\Delta E_{dis}^{H_{2}}$ (per molecule), are calculated as

6 $$\Delta E_{dis}^{clu}=\frac{E_{tot}^{clu\;frozen}-E_{tot}^{clu}}{n+m}$$

and

7 $$\Delta E_{dis}^{H_{2}}=E_{tot}^{H_{2}frozen}-E_{tot}^{H_{2}}$$

where *E*_tot_^clu frozen^ and $E_{tot}^{H_{2}frozen}$ represent the total energies of the frozen sub-nanoclusters and H_2_ molecules, respectively, at their original positions within the H_2_/clu system. These values indicate the energy required to distort configurations from their initial to adsorbed stages. Furthermore, we define the binding energy per atom of the adsorbed system, *E*_b,ads_, as

8 $$E_{b,ads}=\frac{E_{tot}^{H_{2}/clu}-2E_{tot}^{H}-nE_{tot}^{Cu}-mE_{tot}^{Pt}}{2+n+m}=\frac{2E_{b}^{H_{2}}+\left(n+m\right)\left(E_{b}^{clu}+\Delta E_{dis}^{clu}\right)+\Delta E_{\mathrm{int}}+\Delta E_{dis}^{H_{2}}}{2+n+m}$$

This equation incorporates the main energy terms, comprehensively measuring the system’s binding energy.

For structural analyses, we employed the Visualization for Electronic and Structural Analysis (VESTA) software,^52^ in addition to properties such as the average bond length, *d*_av_, and the effective coordination number, ECN, derived from the effective coordination concept.^53,54^ The values of *d*_av, ads_ and ECN_ads_ were obtained for the sub-nanoclusters after adsorption, with molecules subsequently removed from the analyses. The relative deviation, or percentage difference between structural values before and after adsorption, denoted as Δ*d*_av_ and Δ*E*CN, respectively, are given by

9 $$\Delta d_{av}=\frac{\left(d_{av,ads}-d_{av}\right)\times 100}{d_{av}}$$

and

10 $$\Delta ECN=\frac{\left({ECN}_{ads}-ECN\right)\times 100}{ECN}$$

These represent the percentage expansion or contraction of the sub-nanocluster with molecular adsorption and relative percentage coordination. We also considered the equilibrium bond length distances of H–H molecules and the minimum cluster-molecule distance, *d*_Clu‑mol_, using VESTA software. To estimate the alteration in the molecular bond length upon adsorption, we calculated the changes before and after adsorption using the equation:

$$\Delta d_{H-H}=\frac{\left(d_{H-H,ads}{-d}_{H-H}\right)\times 100}{d_{H-H}}$$ 11

Furthermore, electronic and charge distribution analyses were conducted to elucidate our systems’ electronic properties and interaction mechanisms. We considered the local density of states (LDOS) for electronic properties, while the charge analysis was based on a mechanism proposed by Bader.^55^ This method involves partitioning the atomic region into volumes known as Bader volumes, *V*_Bader_, based on charge density. The zero-flow surface, *S*(**r**_*s*_),^56^ is defined as a surface where the electron density gradient, ∇*n*(**r**_*s*_), is perpendicular to the surface, i.e., it is defined as a surface where the electron density, *n*(**r**_*s*_), is a minimum, such that

12 $$\nabla n\left(r_{s}\right)\cdot S\left(r_{s}\right)=\nabla n\left(r_{s}\right)\cdot \mathrm{n̂}\left(r_{s}\right)=0$$

where $\mathrm{n̂}\left(r_{s}\right)$ is the unit normal vector to the surface at point **r**_*s*_. This implies that *S*(**r**_*s*_) is a surface of minimum electron density flow, effectively separating regions of electron density into distinct Bader volumes. In other words, *S*(**r**_*s*_) serves as the boundary surface of a Bader volume, *∂V*_Bader_. The volume *V*_Bader_ = *V*_α,*s*_ around the atomic site α possesses an associated charge density, represented as

13 $$Q_{\alpha }^{Bader}=Z_{\alpha }-\int_{V_{\alpha ,s}}n\left(r_{s}\right)\;d^{3}r_{s}$$

where *Z*_α_ denotes the valence of the atomic site α. Various approaches can determine the volumes *V*_Bader_, with charges optimized through topologies such as Voronoi polyhedra.^57^

## 3 Results and Discussion

All calculations presented in this section were performed using the DFT-PBE+D3 protocol, which has been widely validated for the description of transition-metal nanoclusters, as demonstrated in our previous studies.^23,42,46,47,50,58^ To verify the appropriateness of the PBE functional, we conducted additional tests employing the Tao-Perdew-Staroverov-Scuseria (TPSS) meta-generalized gradient approximation (meta-GGA),^59^ which has been shown to be reliable in describing transition metals.^60,61^ Additionally, relativistic effects, such as spin–orbit coupling (SOC), can be significant for heavy transition metals like Pt, influencing the relative total energies. Thus, we performed supplementary calculations using the TPSS+D3 and PBE+D3+SOC protocols, comparing these results to those obtained with PBE+D3. The outcomes of these tests are provided in the SI (Figure S2 with Table S5, and Figure S3 with Table S6, respectively). While these tests are limited, they confirm the suitability of the PBE+D3 protocol for our systems, though its limitations and the need for cautious interpretation are acknowledged.

### 3.1 Sub-nanocluster Characterization

The lowest energy structures for Cu_*n*_ (*n* = 2–14) sub-nanoclusters as depicted in Figure 1(a) have been verified as true local minima, confirmed for each size through vibrational mode analysis. In this analysis, all the lowest energy configurations exhibited only positive (real) vibrational frequencies (see Figure S1­(a)). The structural patterns and associated property trends of these putative global minimum structures (as obtained from our scalar-relativistic DFT-PBE+D3 protocol) are in complete alignment with previous DFT-PBE studies.^23,42^ Essentially, the growth pattern of Cu sub-nanoclusters follows a bias toward triangular units, where planar structures are preferred up to 6 atoms. Between 6 and 7 atoms, there is a transition from bi- to three-dimensional structures, as a triangular planar structure (Cu_6_) transforms into a close-packed pentagonal bipyramid (Cu_7_) with tetrahedral bonds. The Cu_7_ motif serves as the basic structure for larger sizes, with additional capped and merged pentagonal bipyramids forming polytetrahedrons.

The characterization of Cu sub-nanoclusters, as indicated by the *E*_b_^clu^, *d*_av_, ECN, and total magnetic moment (*m*_tot_) as a function of the number of atoms, *n*, is presented in Figure S4. The magnitude of *E*_b_^clu^ increases with increasing *n*, approaching the cohesive energy for the Cu bulk system (−3.49 eV).^62^ The results for *d*_av_ and ECN can be interpreted through the bond-order-length-strength correlation,^63,64^ which suggests that lower-coordinated (higher-coordinated) atoms tend to have contracted (elongated) bonds, resulting in enhanced (decreased) binding energy. The deviation from monotonic behavior observed for *d*_av_ and ECN between 6 and 7 atoms can be attributed to the transition between planar and globular growth patterns. Regarding *m*_tot_, we observe the characteristic even–odd alternation typical of coinage-metal sub-nanoclusters.^42,47^ This alternation arises from spin polarization occurring in the delocalized *s*-like orbitals, which is directly associated with the Cu valence configuration, specifically, the presence of an opened *s* shell (4*s* ^1^) and filled *d* states (3*d* ^10^). Consequently, sub-nanoclusters with an odd number of atoms exhibit a total magnetic moment of *m*_tot_ = 1.0 μ_B_ (indicative of one unpaired electron), while those with an even number of atoms exhibit *m*_tot_ = 0.0 μ_B_.

We utilized Δ^2^ *E* to ascertain the most stable sizes of Cu_*n*_ within the range of *n* = 2–14, considering the relative stability of each sub-nanocluster size with its neighboring sizes. As depicted in Figure 1(b), the presence of positive peaks enabled us to identify Cu sub-nanoclusters with an even number of atoms (4, 6, 8, 10, and 12) as the most energetically favorable sizes, consistent with prior research.^23,42^ From these specific sub-nanoclusters, we explored the substitution of a single Cu atom with a Pt atom. To achieve this, we subjected all nonequivalent Cu atoms from each lowest energy size sub-nanocluster to structural optimization calculations, aiming to identify the lowest energy Cu_*n*–1_Pt configuration. Figure 1(c) presents the most stable configurations of Cu_*n*_ alongside their corresponding lowest-energy Cu_*n*–1_Pt sub-nanoclusters. The stability of these structures was confirmed through vibrational mode analysis, which showed that all lowest-energy Cu and CuPt configurations exhibited only positive (real) vibrational frequencies (see Figure S1­(b)). Additionally, thermodynamic stability was verified via AIMD simulations, as detailed in Figure S5. These simulations demonstrated that the most stable configurations remained structurally stable at room temperature, with properties before and after the AIMD simulations remaining consistent, as summarized in Table S7. Furthermore, a comparative analysis of the properties of Cu_*n*_ and Cu_*n*–1_Pt sub-nanoclusters, including |*E*_b_|, *d*_av_, ECN, and *m*_tot_, as a function of *n*, is provided in Figure 2.

**Figure 2:**
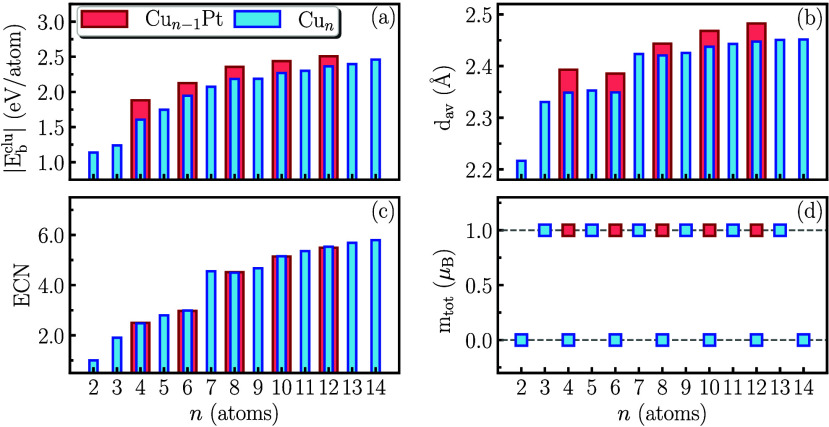
Comparison between Cu_*n*_ and Cu_*n*–1_Pt properties: (a) the magnitude of the binding energy (|*E*_b_^clu^|), (b) the average bond length (*d*_av_), (c) the effective coordination number (ECN), and (d) the total magnetic moment (*m*_tot_), as a function of the atoms number (*n*).

Qualitatively, the same structural motifs are found for both Cu_*n*_ and Cu_*n*–1_Pt systems (see Figure 1(c)). Substituting a Cu atom with a Pt atom does not alter the overall geometry of the sub-nanoclusters. However, as shown in Figure 2(a), the inclusion of a Pt atom increases the magnitude of *E*_b_^clu^ by between 6.1% (Cu_11_Pt) and 17.1% (Cu_3_Pt). This stabilization can be attributed to the higher cohesive energy of Pt (5.84 eV per atom) compared to Cu (3.49 eV per atom).^62^ Correspondingly, due to the larger atomic radius of Pt (1.39 Å) compared to Cu (1.28 Å),^62^ there is a 0.9% to 1.9% increase in the average bond length (*d*_av_) of CuPt sub-nanoclusters relative to their Cu counterparts (Figure 2(b)). In terms of geometry reflected in the ECN (Figure 2(c)), minimal changes were observed between the two sets. Finally, the well-established oscillatory zigzag behavior of the total magnetic moment (*m*_tot_) is altered (Figure 2(d)). The removal of a Cu atom causes *m*_tot_ to shift from 0 to 1.0 μ_B_, indicating that the dominant magnetic properties are still primarily influenced by the majority Cu atoms, with the Pt atom contributing to the electron density pool of the sub-nanocluster.

Given the size of our sub-nanoclusters, the electronic character is within the molecular limit, resulting in a quasi-discrete DOS, as shown in Figure S6. For both Cu_*n*_ and Cu_*n*–1_Pt systems, the total and local DOS of the occupied states is dominated by *d*-states, which is typical for transition-metal systems. This dominance increases with the number of atoms in the sub-nanoclusters due to the broadening of the *d*-states with increased coordination number. Additionally, the LDOS is sensitive to the atomic structure. Despite Cu_*n*_ and Cu_*n*–1_Pt maintaining the same geometric motif, the substitution of one Cu atom with a Pt atom breaks the structural symmetry. This symmetry breaking results in a broader LDOS for Cu_*n*–1_Pt sub-nanoclusters compared to the narrower LDOS for Cu_*n*_ sub-nanoclusters. The Cu_*n*_ sub-nanoclusters exhibit many degenerate states, reflected in the well-defined, numerous sharp peaks in the LDOS. In contrast, Cu_*n*–1_Pt systems show a more spread out LDOS due to the introduction of Pt breaking the symmetry and thus reducing the degeneracy.

The trends observed in the average bond lengths and binding energies of the Cu_*n*–1_Pt sub-nanoclusters can be attributed to the coordination environment of the Pt atom and the resulting strain on the sub-nanocluster. In smaller sub-nanoclusters like Cu_3_Pt, the lower coordination around the Pt atom results in weaker bonding interactions, reflected in larger average bond lengths and lower binding energy magnitudes. For Cu_5_Pt, the increased coordination enhances Pt– Cu bonding, leading to shorter bond lengths and higher binding energy magnitudes. This trend persists until Cu_7_Pt, where geometric factors become more dominant as the sub-nanocluster size increases. The LDOS analysis in Figure S6 corroborates these findings, showing stronger hybridization between Pt *d*-states and Cu states for Cu_5_Pt compared to Cu_3_Pt. This enhanced hybridization explains the stronger bonding and higher binding energy magnitudes observed for Cu_5_Pt.

The presence of Pt in the Cu sub-nanoclusters significantly influences the electronic structure, primarily by intensifying the *d*-state contributions around the Fermi level in the LDOS. This effect is evident from the qualitative charge density plots (Figure S7) and the increased isosurface concentration around Pt atoms, highlighted in the charge density difference plots (Figure S8). These analyses show that the charge density in pure Cu sub-nanoclusters remains relatively uniform, indicating an equal charge distribution throughout the system. In contrast, CuPt sub-nanoclusters exhibit localized changes in charge density near the Pt atoms.

The introduction of Pt breaks the structural symmetry of the sub-nanoclusters, which is reflected in the LDOS. The magnetization density further confirms this symmetry breaking, as illustrated in Figure S9. The magnetization density analysis reveals the contributions of unpaired spins per atom to the overall magnetization of the sub-nanocluster. All spins are paired in pure Cu sub-nanoclusters, resulting in no net local magnetic moment. However, in CuPt sub-nanoclusters, unpaired spins are observed around the Pt and nearby Cu atoms, indicating that these regions significantly contribute to the sub-nanocluster’s magnetic moment. This confirms that adding a Pt atom not only disrupts the charge distribution but also enhances the magnetic properties of the sub-nanoclusters by introducing unpaired spins.

### 3.2 Molecular Adsorption

After selecting and characterizing the substrates for molecular adsorption, i.e., the lowest energy Cu_*n*_ and Cu_*n*–1_Pt sub-nanoclusters, we confirmed the main properties of the H_2_ molecule: binding energy $\left(E_{b}^{H_{2}}\right)$ , equilibrium bond length (*d*_H–H_), and vibrational frequency (ν). Specifically, we obtained $E_{b}^{H_{2}}=-2.27eV$ , *d*_H–H_ = 0.75 Å, and ν = 4328 cm^–1^, which are in excellent agreement with experimental data.^65,66^ To provide a more accurate total energy for H_2_, including the zero-point energy (ZPE) reflecting the vibrational energy of the quantum mechanical ground state, we recalculated the H_2_ molecule’s energy. This calculation was performed at 1 atm pressure and 300 K, incorporating the Gibbs free energy. The ZPE value obtained is 0.268 eV, aligning well with the experimental value of ≈0.270 eV reported by Irikura.^67^

Next, we investigated H_2_ adsorption on all nonequivalent top, bridge, and hollow sites of the Cu_*n*_ and Cu_*n*–1_Pt sub-nanoclusters (*n* = 4, 6, 8, 10, and 12), leading to the formation of H_2_/Cu_*n*_ and H_2_/Cu_*n*–1_Pt systems, considering both H_2_-bonding modes: side-on and end-on.^49^ The resulting lowest energy configurations are shown in Figure 3. For all sizes of Cu_*n*_ sub-nanoclusters, H_2_ preferentially adsorbs on the Cu top (1-fold) sites, where each H atom interacts with a Cu atom. In this case, the lowest energy H_2_Cu_*n*_ configurations are characterized by side-on motifs, with both H atoms aligned parallel to the Cu_*n*_ sub-nanoclusters (dihydrogen binding). In contrast, for Cu_*n*–1_Pt sub-nanoclusters, H_2_ prefers to bind at the Pt top sites, resulting in each H atom interacting with a Pt atom after structural optimization. Consequently, the lowest energy H_2_/Cu_*n*–1_Pt configurations lead to H_2_ dissociation. While we did not specifically test for the dissociation mode of H_2_, it emerges as the result of our structural optimizations. The inclusion of the Pt atom introduces significant changes compared to Cu-only sub-nanoclusters, such as an increased equilibrium distance within the H_2_ molecule, weakening the H–H bond and potentially causing chemical dissociation, leading to hydridic-like bonding (see our discussion on charge analysis).

**Figure 3:**
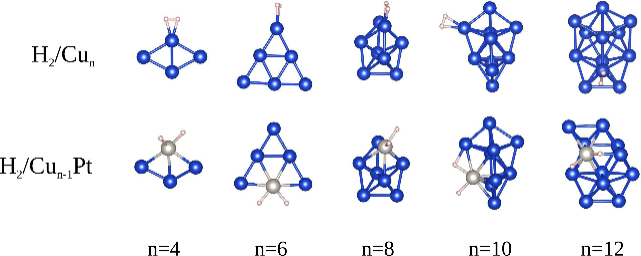
Lowest energy H_2_/Cu_*n*_ and H_2_Cu_*n*–1_Pt systems, where *n* = 4, 6, 8, 10, and 12.

Notably, for *n* = 10, we observe H atoms binding to both Cu and Pt, indicating the potential breakage of the H_2_ molecular bond due to the influence of the Pt atom. Additionally, for *n* = 12, we observe significant structural distortion triggered by H_2_ adsorption on the Pt atom. These findings underscore the significant impact of Pt substitution on the adsorption behavior and structural stability of Cu sub-nanoclusters, revealing enhanced interactions and potential chemical activation sites due to the presence of Pt.

To deepen our understanding of adsorbed systems, we calculated several relevant properties: |*E*_ads_|, *d*_Clu‑mol_, Δ*d*_av_, Δ*E*CN, *m*_tot_, Δ*d*_H–H_ upon molecular adsorption as a function of *n*. These adsorption properties are depicted in Figure 4 for the lowest energy H_2_/Cu_*n*_ systems, which retain the H–H bond (w/ H–H); the most stable state among those configurations with relatively higher energy level H_2_/Cu_*n*–1_Pt systems, which also retain the H–H bond (w/ H–H); and the lowest energy H_2_/Cu_*n*–1_Pt systems, which do not retain the H–H bond (w/o H–H). In Figure 4(a), the |*E*_ads_| trend is shown. Since adsorption energy measures the magnitude of the binding energy of the H_2_ molecule to the sub-nanocluster, we can see that CuPt sub-nanoclusters have higher |*E*_ads_| values, indicating a stronger nanocluster-molecule interaction. This underscores the significant role of the Pt atom in the Cu-based sub-nanoclusters. The highest adsorption energy magnitudes occur for the smallest sub-nanoclusters (*n* = 4), while the lowest occur for the largest (*n* = 12). Sub-nanoclusters with *n* = 6, 8, and 10 atoms exhibit intermediate values that follow this trend consistently.

**Figure 4:**
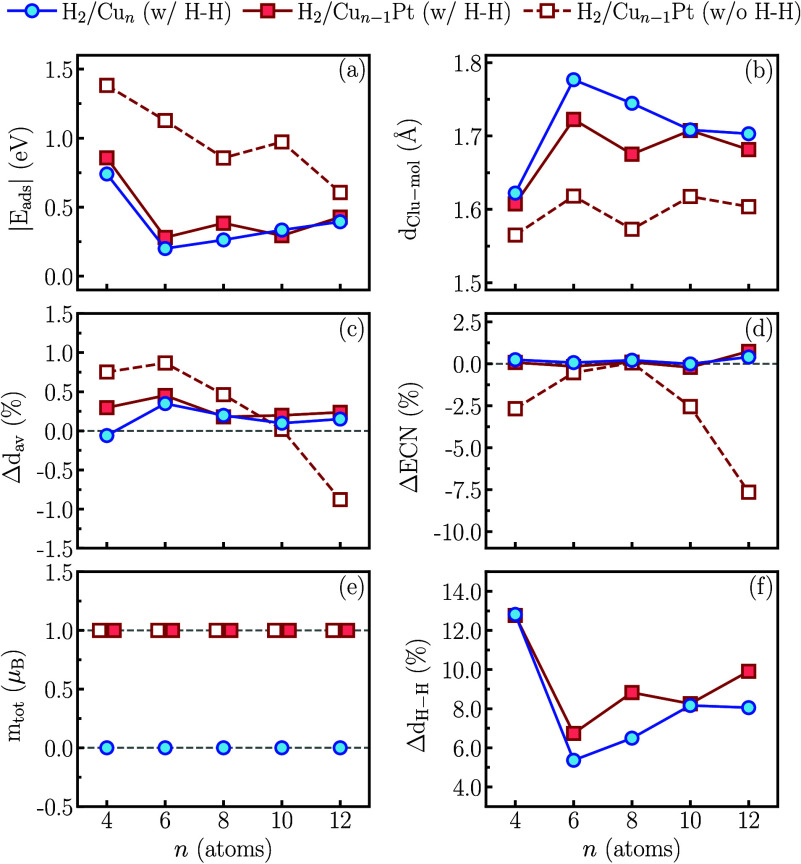
Comparison between H_2_/Cu_*n*_ and H_2_/Cu_*n*–1_Pt properties: (a) adsorption energy magnitude (|*E*_ads_|), (b) minimum cluster-molecule distance (*d*_Clu‑mol_), (c) relative deviation for *d*_av_ (Δ*d*_av_) upon molecular adsorption, (d) relative deviation for ECN (Δ*E*CN) upon molecular adsorption, (e) total magnetic moment (*m*_tot_), and (f) relative deviation for *d*_H–H_ (Δ*d*_H–H_) upon molecular adsorption, as a function of the number of atoms (*n*), where *n* = 4, 6, 8, 10, and 12. Data in blue filled circles (continuous line) describe the lowest energy H_2_/Cu_*n*_ systems where there is a H–H bond (w/ H–H); data in red filled squares (continuous line) describe the most stable higher energy H_2_/Cu_*n*–1_Pt systems where there is a H–H bond (w/ H–H); data in red empty squares (dashed line) describe the lowest energy H_2_/Cu_*n*–1_Pt systems where there is not a H–H bond (w/o H–H).

Even when considering higher energy H_2_/CuPt systems that maintain the H–H bond, the most stable isomers after adsorption are still more energetically favorable than H_2_/Cu systems, with one exception at *n* = 10 (practically degenerate at 0.04 eV). Consequently, this result reveals that the presence of Pt not only intensifies the interaction but also naturally promotes the breaking of the H_2_ bond due to the significant distance between the H atoms in these systems. Our results for the *E*_ads_ concerning H_2_/Cu_*n*_ systems are in good agreement with the literature,^26^ while the findings for H_2_/Cu_*n*–1_Pt corroborate the beneficial synergistic effects of combining Cu and Pt.^58^

Comparing the adsorption energies (*E*_ads_) of H_2_ on CuPt (w/o H–H) and Cu (w/ H–H) systems, we observe that the presence of Pt and its interaction with H_2_ generally intensifies the interaction by at least 0.5 eV for most cluster sizes. However, for *n* = 12, the shift in adsorption energy is only 0.2 eV. This slight decrease is due to part of the interaction energy being directed toward the structural change of the sub-nanocluster, altering its geometry in the region close to the adsorption site. The stronger interaction of the molecule with the CuPt (w/o H–H) systems compared to Cu (w/ H–H) and CuPt (w/ H–H) systems is supported by the closer proximity of the molecule to the sub-nanocluster, as shown by *d*_Clu‑mol_ in Figure 4(b).

The greater nanocluster-molecule interaction from a structural standpoint is evident in the higher values of relative deviation given by Δ*d*_av_ and Δ*E*CN upon molecular adsorption for PtCu relative to Cu systems, as shown in Figure 4(c) and (d), respectively. While purely Cu systems (and CuPt w/ H–H) exhibit almost constant relative deviation, indicating a slight expansion of the sub-nanocluster bonds after adsorption and unchanged coordination of the sub-nanoclusters, systems with CuPt (w/o H–H) show a greater expansion of *d*_av_ as the size of the sub-nanoclusters decreases. This expansion transforms into a contraction and compaction for *n* = 12, reflecting the change in structural motif due to adsorption and resulting in a decrease in coordination assumed by the new distorted structure.

In terms of magnetic properties, we observed from Figure 4(e) that the adsorption of H_2_ does not significantly alter the spin configuration to the extent of changing the *m*_tot_ values. The magnetic moment trend continues to be dictated by the electronic character of Cu atoms for the adsorbed systems, where we have *m*_tot_ = 0.0 μ_B_ or 1.0 μ_B_ for clusters with even or odd numbers of Cu atoms, respectively. However, in electronic terms, we observed a greater influence of H_2_ adsorption on the sub-nanoclusters in the LDOS, as depicted in Figure S10. Due to the limited number of reactive sites in finite sub-nanoclusters, the adsorption of just one molecule onto the sub-nanocluster surface is adequate to reduce the highest density of states in the region close to the Fermi level. This effect is illustrated in Figure S11, where we compare the center of gravity of the occupied *d*-states (ε_*d*_)^68^ for the sub-nanoclusters before and after adsorption. The ε_*d*_ can be associated with the adsorption energy of H_2_, which can indirectly influence the reactivity of the systems; the closer the ε_*d*_ approaches the Fermi level, the more reactive they become. Notably, the ε_*d*_ for Cu is closer to the Fermi level than the ε_*d*_ for Pt *d*-states, even though Cu *d*-states are filled. This observation can be explained by the localized nature of the Cu 3*d*-states.

Finally, Figure 4(f) shows the relative deviation in the equilibrium distance of the H_2_ molecule upon adsorption, compared to its value in the gas phase (0.75 Å). Notably, the values of Δ*d*_H–H_ are presented for Cu (w/ H–H bond) and CuPt (w/ H–H bond), representing cases where the H–H bond remains intact. This comparison is crucial because, for the most stable H_2_/CuPt configurations, H_2_ dissociates. Adsorption on Cu_*n*_ sub-nanoclusters increases the H–H bond distance compared to the gas-phase molecule, with the distance increasing further until the molecule dissociates on Cu_*n*–1_Pt systems. The distances between H atoms range from 1.87 Å for *n* = 4 to 2.27 Å for *n* = 12, indicating a very weak interaction between the H atoms. This result is corroborated by Figure S12, which shows a significant decrease in the vibrational frequency values, from the gas-phase H_2_ molecule to the Cu-based sub-nanoclusters, and further to the CuPt-based sub-nanoclusters, where only a very weak chemical bond exists between the H atoms. The lowest ν values between H atoms on CuPt, relative to Cu, are fully consistent with the highest *E*_ads_ magnitudes and *d*_H–H_ values. Furthermore, the most stable configurations, dihydrogen (H_2_ side-on) adsorption for Cu_*n*_ sub-nanoclusters and H_2_ dissociation for Cu_*n*–1_Pt sub-nanoclusters, are further supported by the estimation of activation and reaction energies, as presented in Figure S13. Additionally, the atomic configurations for H_2_/Cu_*n*–1_Pt sub-nanoclusters, considering the most stable higher-energy H_2_/Cu_*n*–1_Pt systems where the H–H bond is intact (w/ H–H), are presented in Figure S14, along with the corresponding relative total energies compared to the lowest-energy H_2_/Cu_*n*–1_Pt configurations (w/o H–H).

### 3.3 Interaction Mechanism

To enhance our understanding of the enhanced interaction between Cu_*n*–1_Pt and H_2_ facilitated by the Pt atom, we conducted detailed energetic analyses. First, in Figure 5(a), we present a comparative analysis of the binding energies of the two sub-nanocluster compositions using eq 3 to calculate Δ*E*_b_^clu^. Both compositions exhibit stability with negative binding energies. However, a clear energetic preference for Cu_*n*–1_Pt sub-nanoclusters over Cu_*n*_ sub-nanoclusters for the same *n* values is observed. Furthermore, the greater stabilization is more pronounced in smaller sub-nanoclusters due to the presence of the Pt atom. Specifically, we find values of Δ*E*_b_^clu^ following the order: 17.1% for *n* = 4, 9.3% for *n* = 6, 7.9% for *n* = 8, 7.5% for *n* = 10, and 6.1% for *n* = 12.

**Figure 5:**
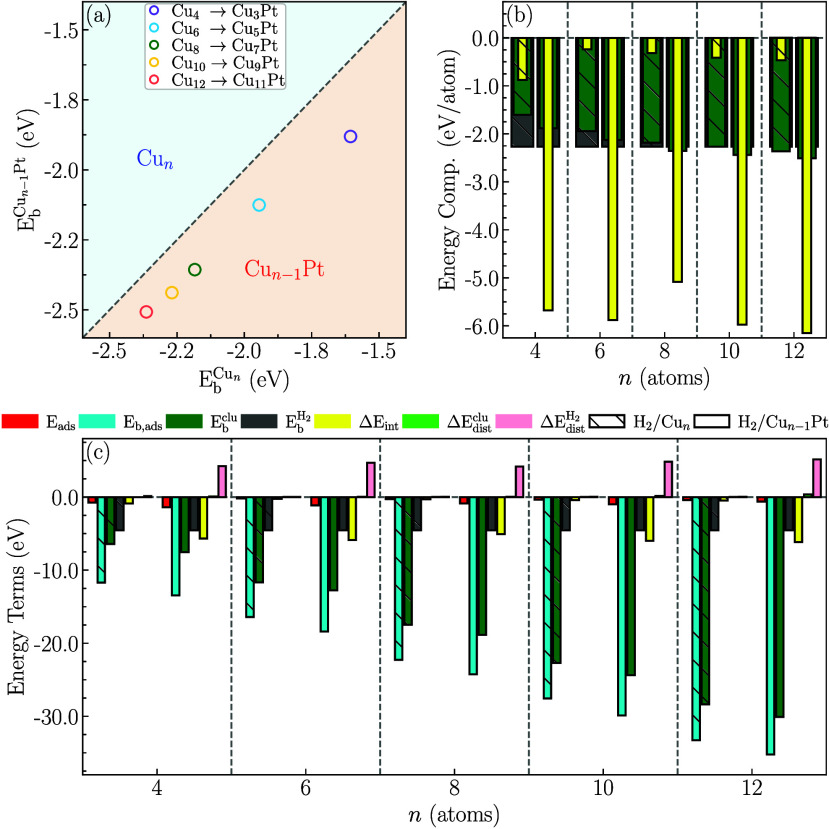
Energetic analysis: (a) relative binding energy, involving Δ*E*_b_^clu^ for comparing sub-nanoclusters; (b) energy competition among $E_{b}^{H_{2}}$ , *E*_b_^clu^, and Δ*E*_int_ (per atom); and (c) the main energy terms for the energetic analysis, considering the adsorption (*E*_ads_) and binding (*E*_b,ads_) energies for the lowest energy H_2_/clu systems.

In principle, the higher stability observed for CuPt sub-nanoclusters might suggest lower reactivity toward the H_2_ molecule. However, our analysis in Figure 5(b) reveals the competition among the main additive energy terms of the adsorbed systems (see eq 8). For the H_2_/Cu_*n*_ systems, the highest energetic contribution (in absolute value) originates from the binding energy of the molecule (−2.27 eV), followed by that of the sub-nanocluster (ranging from −1.61 eV for *n* = 4 to −2.36 eV for *n* = 12), with the H_2_-clu interaction energy being the third in magnitude (from −0.88 eV for *n* = 4 to −0.47 eV for *n* = 12). For the H_2_/Cu_n-1_Pt systems, we observe a significantly intensified interaction energy due to the presence of the Pt atom, with Δ*E*_int_ ranging from −5.09 to −6.15 eV. Here, $E_{b}^{H_{2}}$ contributes the second largest magnitude, while *E*_b_^clu^ ranks third (from −1.88 eV for *n* = 4 to −2.51 eV for *n* = 12). Consequently, we have demonstrated that, apart from being more stable, CuPt-based sub-nanoclusters are also the most reactive, exhibiting high interaction energy values with H_2_.

In Figure 5(c), we present a comprehensive overview of the energy term contributions that govern the definitions of *E*_ads_ (eq 4) and *E*_b,ads_ (eq 8). Essentially, we observe that all terms involved in these equations are additive (account for negative energy values), except for the distortion energy terms ( $\Delta E_{dis}^{H_{2}}$ and Δ*E*_dis_^clu^), which represent penalties or positive energy terms. These distortion energies reflect energy losses due to structural adjustments arising from adsorption, leading to changes in the configurations of the sub-nanoclusters and the molecule from their lowest energy structures. In this context, we can correlate the values of $\Delta E_{dis}^{H_{2}}$ and Δ*E*_dis_^clu^ with the Δ*d*_av_, Δ*E*CN, and Δ*d*_H–H_ values. We observe minimal structural penalties for most systems, except for molecules adsorbed in CuPt-based systems, which exhibit high $\Delta E_{dis}^{H_{2}}$ values. This is attributed to the dissociation (breaking of H–H bonds) of these molecules due to the substantial interaction (Δ*E*_int_) with CuPt-based sub-nanoclusters.

Combining the insights from Figure 5(b) and (c), we can delineate the mechanism underlying H_2_ adsorption on the sub-nanoclusters. In the H_2_/Cu_*n*_ scenario, the binding energies of the sub-nanocluster and the molecule overlap the interaction energy, indicating a typical adsorption process characterized by moderate interaction, which allows for potential molecular desorption. This suggests that the sub-nanoclusters and the molecules maintain structural and constitutional integrity. However, for the H_2_/Cu_n-1_Pt systems, we observe a distinct scenario. Here, the interaction with the Pt atom plays a pivotal role by providing an interaction energy that exceeds the binding energies of the sub-nanocluster and the molecule. Furthermore, the binding energy of the cluster surpasses that of the molecule. Consequently, the H_2_–Cu_n-1_Pt interaction is sufficiently strong to induce molecule dissociation initially, observed across all studied sub-nanocluster sizes, and subsequently lead to greater distortions in the sub-nanoclusters, as evidenced in the case of *n* = 12.

In Figure 6, our energy analysis is complemented by effective Bader charge analysis, where the charge transfer is calculated as Δ*Q*_Bader_ = *Z*_val_ – *Q*_Bader_, with *Z*_val_ representing the number of valence electrons and *Q*_Bader_ the Bader charge, for the most stable H_2_/clu systems. A minimal charge exchange indicates weak interaction between H_2_ and Cu_*n*_ sub-nanoclusters, whereas a pronounced charge exchange is observed in H_2_ and Cu_*n*–1_Pt systems, which reflects a net charge transfer from the CuPt sub-nanocluster (cationic) to the H atoms (anionic). As noted by Lamanec et al.,^69^ a standard hydrogen bond involves a protonic hydrogen (with a partial positive charge) interacting with an electron donor, similar to the dihydrogen binding motifs in H_2_/Cu_*n*_. In contrast, the H_2_/Cu_*n*–1_Pt system resembles a hydridic hydrogen bond, where a hydridic hydrogen (with a partial negative charge) interacts with an electron acceptor, albeit with some reservations.

**Figure 6:**
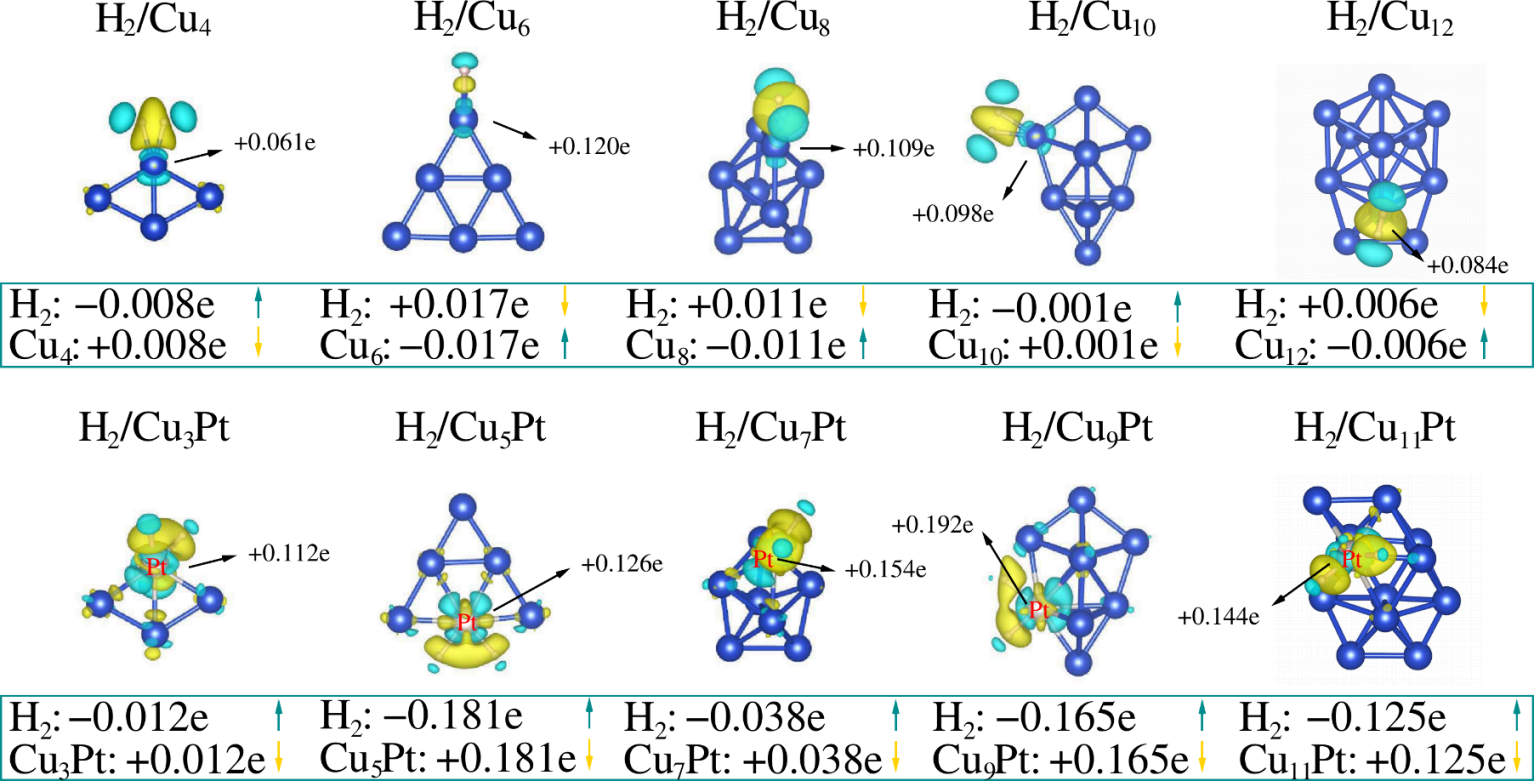
Bader charge flow, Δ*Q*_Bader_, for H_2_/Cu_*n*_ and H_2_/Cu_*n*–1_Pt systems. The yellow isosurface represents charge density losses per atom, while the cyan isosurface represents corresponding gains per atom. Effective charge exchanges between sub-nanoclusters and molecules are also depicted in nominal values, in units of *e*. The Δ*Q*_*Bader*_ values of the adsorption site atoms are highlighted in the figure. In all cases, the isosurfaces are set to 0.005.

Our findings on H_2_ adsorption on Cu and CuPt sub-nanoclusters corroborate well-established knowledge regarding the different chemical nature of these binding situations.^70–72^ The dihydrogen binding motifs in the H_2_/Cu_*n*_ sub-nanoclusters represent a weak interaction, where the H_2_ molecule interacts with a metal surface atom from Cu_*n*_, a site capable of accepting electron density. This interaction typically involves charge transfer from the metal into the H–H antibonding orbital, leading to some back-donation and slight weakening of the H–H bond without breaking it. The H_2_ molecule retains its molecular form, and the bonding is primarily physisorption or weak chemisorption. This interaction is not a hydridic hydrogen bond because the H_2_ molecule remains intact, with no evidence of hydridic or protic character in the individual hydrogen atoms.

In contrast, in the H_2_/Cu_*n*–1_Pt system, the H_2_ molecule dissociates, resulting in two H atoms adsorbed on the sub-nanocluster. This occurs because the Pt atom introduces stronger interaction between the molecule and the sub-nanocluster, activating the H–H bond breaking, facilitated by the electronic properties of Pt, which has a higher ability to stabilize the resulting H atoms via chemisorption (due to its higher affinity for H). This activation is supported by our Bader charge analysis, where substantial charge transfer from the sub-nanocluster to the hydrogen molecule occurs. The Pt atom draws electron density from the sub-nanocluster, enabling the H–H bond to break and leaving two adsorbed H atoms. The resulting system involves metal–hydrogen bonds, with the individual H atoms chemisorbed to the sub-nanocluster surface. Hydric bonding typically involves a hydride (H−) species forming an attractive interaction with an electrophilic center. In the case of H_2_/Cu_*n*–1_Pt, the dissociated H atoms are negatively charged upon adsorption, but they do not act as hydrides. Instead, they are individually chemisorbed, with their bonding dominated by localized metal–hydrogen interactions. The positive charge on the CuPt sub-nanocluster indicates that the sub-nanocluster is donating electron density, likely facilitated by the Pt atom, which exhibits higher catalytic activity and a strong affinity for adsorbates like H_2_. While electron density transfer creates a partial hydridic character in H_2_, the bonding mechanism is more accurately described as H_2_ dissociation, forming direct metal–hydrogen bonds. For hydridic bonding, negative charge would need to be predominantly localized on the hydrogen atoms (not on the intact H_2_ molecule), with weak interactions between hydridic hydrogens and electrophilic species, such as secondary interactions rather than strong chemisorption. Therefore, the charge transfer observed in the Bader analysis suggests a strong interaction with partial hydridic character. In essence, the substitution of a Cu atom with a Pt atom enhances electron density flow from Cu to Pt. Finally, Table S8 presents the hybridization indices, supporting our interaction mechanism, showing larger *sd* hybridization and negligible *sp* hybridization for the systems.

## 4 Conclusions

Our DFT-PBE+D3 calculations provide valuable insights into the intricate interactions between H_2_ molecules and both Cu_*n*_ and Cu_*n*–1_Pt sub-nanoclusters. We elucidated the adsorption behavior and underlying mechanisms governing these interactions through rigorous computational simulations and energetic analyses. Initially, we conducted a thorough investigation of Cu_*n*_ sub-nanoclusters (*n* = 2–14) as potential substrates for adsorption. This involved comprehensive energetic, structural, and electronic characterizations, culminating in the selection of the lowest energy sizes (4, 6, 8, 10, and 12) using a stability function. Subsequently, we explored the substitution of a single Cu atom with a Pt atom in the context of SAA sub-nanoclusters, resulting in the formation of Cu_*n*–1_Pt sub-nanoclusters. Despite structural similarities between Cu_*n*_ and Cu_*n*–1_Pt systems, the presence of Pt significantly enhanced the stabilization, as indicated by the binding energy and corroborated by vibrational frequency analyses and AIMD simulations. Upon identifying the lowest energy adsorption sites for both H_2_/Cu_*n*_ and H_2_/Cu_*n*–1_Pt systems, we observed distinct behaviors. While Cu-only sub-nanoclusters exhibited a H_2_-bonding mode known as H_2_ side-on, with weak interactions and minimal charge exchange, the SAA sub-nanoclusters displayed a different H_2_-bonding mode, characterized by molecular dissociation. This resulted in strong interactions, marked by substantial charge transfer from the sub-nanoclusters to the H_2_ molecules. The presence of Pt not only intensified the interaction but also facilitated the breaking of the H_2_ bond. This effect was further underscored by significantly enhanced interaction energies in CuPt-based systems (interaction energy magnitude from 5.09 to 6.15 eV), surpassing those in Cu-only systems (interaction energy magnitude from 0.47 to 0.88 eV). In the H_2_/Cu scenario, the binding energies of the sub-nanocluster and the molecule overlapped with the interaction energy, suggesting a typical adsorption process with moderate interaction. Conversely, in H_2_/CuPt systems, the interaction with Pt atoms exceeded the binding energies of both the sub-nanocluster and the molecule, leading to molecular dissociation and subsequent distortions in the sub-nanoclusters. Our findings highlight the pivotal role of Pt atoms in enhancing the interaction between H_2_ molecules and sub-nanocluster substrates. The presence of Pt introduces significant energetic modifications, resulting in intensified adsorption energies and altered charge transfer dynamics. Notably, SAA CuPt-based sub-nanoclusters exhibited clear preferences over pure Cu counterparts in terms of stability and reactivity toward H_2_ adsorption, underscoring the synergistic effects arising from the Cu– Pt combination. In addition to our computational approaches, we recognize the potential of machine learning (ML) algorithms in exploring more extensive and more complex sub-nanocluster systems. By leveraging advanced ML techniques, such as the Bayesian Optimization algorithm, it is possible to efficiently navigate the vast configurational space of sub-nanoclusters with more significant numbers of atoms. This approach can identify promising candidates for enhanced H_2_ adsorption and catalytic activity by predicting stable and active sub-nanocluster configurations without exhaustive computational resources. Integrating ML algorithms with our DFT-PBE+D3 calculations could significantly accelerate the discovery and optimization of high-performance nanocatalysts, paving the way for innovative advancements in hydrogen-related technologies.

## Supplementary Material



## Notes

Convergence tests, sub-nanocluster vibrational frequencies, the impact of alternative functionals (TPSS), the relativistic effects of spin–orbit coupling (SOC), sub-nanoclusters properties, *ab initio* molecular dynamics (AIMD) simulations, density of states for sub-nanoclusters, electronic charge analysis, magnetic analysis, density of states for adsorbed systems, center of gravity of the occupied *d* state, H_2_ vibrational frequencies, estimation of activation energy, H_2_/Cu_*n*–1_Pt atomic configuration (w/ H–H), hybridization index, and atomic coordinates (PDF)

## References

[1] Bell A. T. (2003). The Impact
of Nanoscience on Heterogeneous Catalysis. Science.

[2] Friend C. M., Xu B. (2017). Heterogeneous Catalysis:
A Central Science for a Sustainable Future. Acc. Chem. Res..

[3] Li X., Mitchell S., Fang Y., Li J., Perez-Ramirez J., Lu J. (2023). Advances in heterogeneous single-cluster catalysis. Nat. Rev. Chem..

[4] Alonso J. A. (2000). Electronic
and Atomic Structure, and Magnetism of Transition-Metal Clusters. Chem. Rev..

[5] Fernando A., Weerawardene K. L. D. M., Karimova N. V., Aikens C. M. (2015). Quantum
Mechanical Studies of Large Metal, Metal Oxide, and Metal Chalcogenide
Nanoparticles and Clusters. Chem. Rev..

[6] Liu L., Corma A. (2018). Metal Catalysts for
Heterogeneous Catalysis: From Single Atoms to
Nanoclusters and Nanoparticles. Chem. Rev..

[7] Yue M., Lambert H., Pahon E., Roche R., Jemei S., Hissel D. (2021). Hydrogen energy systems:
A critical review of technologies,
applications, trends and challenges. Renewable
Sust. Energy Rev..

[8] Singla M. K., Nijhawan P., Oberoi A. S. (2021). Hydrogen fuel and fuel cell technology
for cleaner future: a review. Environ. Sci.
Pollut. Res..

[9] Hannagan R. T., Giannakakis G., Flytzani-Stephanopoulos M., Sykes E. C. H. (2020). Single-Atom
Alloy Catalysis. Chem. Rev..

[10] Du Y., Sheng H., Astruc D., Zhu M. (2020). Atomically Precise
Noble Metal Nanoclusters as Efficient Catalysts: A Bridge between
Structure and Properties. Chem. Rev..

[11] Janjua M. R. S. A. (2021). Prediction
and Understanding: Quantum Chemical Framework of Transition Metals
Enclosed in a B_12_N_12_ Inorganic Nanocluster for
Adsorption and Removal of DDT from the Environment. Inorg. Chem..

[12] Wang S., Lu A., Zhong C.-J. (2021). Hydrogen
production from water electrolysis: role of
catalysts. Nano Converg.

[13] Tyo E. C., Vajda S. (2015). Catalysis by clusters with precise
numbers of atoms. Nat. Nanotechnol..

[14] Tang Q., Hu G., Fung V., Jiang D. (2018). Insights into interfaces, stability,
electronic properties, and catalytic activities of atomically precise
metal nanoclusters from first principles. Acc.
Chem. Res..

[15] Zhang W., Fu Q., Luo Q., Sheng L., Yang J. (2021). Understanding Single-Atom
Catalysis in View of Theory. JACS Au.

[16] Darby M. T., Stamatakis M., Michaelides A., Sykes E. C. H. (2018). Lonely Atoms
with Special Gifts: Breaking Linear Scaling Relationships in Heterogeneous
Catalysis with Single-Atom Alloys. J. Phys.
Chem. Lett..

[17] Giannakakis G., Flytzani-Stephanopoulos M., Sykes E. C. H. (2019). Single-Atom Alloys
as a Reductionist Approach to the Rational Design of Heterogeneous
Catalysts. Acc. Chem. Res..

[18] Bunting R. J., Wodaczek F., Torabi T., Cheng B. (2023). Reactivity of Single-Atom
Alloy Nanoparticles: Modeling the Dehydrogenation of Propane. J. Am. Chem. Soc..

[19] Liu X., Astruc D. (2018). Atomically precise copper nanoclusters and their applications. Coord. Chem. Rev..

[20] Baghdasaryan A., Bürgi T. (2021). Copper nanoclusters: designed synthesis,
structural
diversity, and multiplatform applications. Nanoscale.

[21] Wu Q.-J., Si D.-H., Sun P.-P., Dong Y.-L., Zheng S., Chen Q., Ye S.-H., Sun D., Cao R., Huang Y.-B. (2023). Atomically Precise Copper Nanoclusters
for Highly Efficient
Electroreduction of CO_2_ towards Hydrocarbons via Breaking
the Coordination Symmetry of Cu Site. Angew.
Chem., Int. Ed..

[22] Chu X., Xiang M., Zeng Q., Zhu W., Yang M. (2011). Competition
Between Monomer and Dimer Fragmentation Pathways of Cationic Cu_n_ Clusters of *n* = 2 – 20. J. Phys. B: At. Mol. Opt. Phys..

[23] Chaves A. S., Rondina G. G., Piotrowski M. J., Tereshchuk P., Da Silva J. L. F. (2014). The Role of Charge States in the
Atomic Structure of
Cu_n_ and Pt_n_ (*n* = 2 –
14 Atoms) Clusters: A DFT Investigation. J.
Phys. Chem. A.

[24] Zhang L., Liu H., Liu S., Banis M. N., Song Z., Li J., Yang L., Markiewicz M., Zhao Y., Li R., Zheng M., Ye S., Zhao Z.-J., Botton G. A., Sun X. (2019). Pt/Pd Single-Atom Alloys as Highly Active Electrochemical Catalysts
and the Origin of Enhanced Activity. ACS Catal..

[25] Zhang X., Cui G., Feng H., Chen L., Wang H., Wang B., Zhang X., Zheng L., Hong S., Wei M. (2019). Platinum-copper
single atom alloy catalysts with high performance towards glycerol
hydrogenolysis. Nat. Commun..

[26] Kuang X.-J., Wang X.-Q., Liu G.-B. (2011). A density functional
study on the
adsorption of hydrogen molecule onto small copper clusters. J. Chem. Sci..

[27] Janjua M. R. S. A. (2022). Hydrogen
as an energy currency: Encapsulation of inorganic Ga_12_N_12_ with alkali metals for efficient H_2_ adsorption
as hydrogen storage materials. J. Phys. Chem.
Solids.

[28] Hohenberg P., Kohn W. (1964). Inhomogeneous Electron
Gas. Phys. Rev..

[29] Kohn W., Sham L. J. (1965). Self-Consistent
Equations Including Exchange and Correlation
Effects. Phys. Rev..

[30] Perdew J. P., Burke K., Ernzerhof M. (1996). Generalized
Gradient Approximation
Made Simple. Phys. Rev. Lett..

[31] Grimme S., Antony J., Ehrlich S., Krieg H. (2010). A Consistent and Accurate
Ab Initio Parametrization of Density Functional Dispersion Correction
(DFT-D) for the 94 Elements H-Pu. J. Chem. Phys..

[32] Grimme S., Hansen A., Brandenburg J. G., Bannwarth C. (2016). Dispersion-Corrected
Mean-Field Electronic Structure Methods. Chem.
Rev..

[33] Blöchl P. E. (1994). Projector
Augmented-Wave Method. Phys. Rev. B.

[34] Kresse G., Joubert D. (1999). From Ultrasoft Pseudopotentials
to the Projector Agumented-Wave
Method. Phys. Rev. B.

[35] Kresse G., Hafner J. (1993). Ab Initio Molecular
Dynamics for Open-Shell Transition
Metals. Phys. Rev. B.

[36] Kresse G., Furthmüller J. (1996). Efficient
Iterative Schemes for Ab Initio Total-Energy
Calculations Using a Plane-Wave Basis Set. Phys.
Rev. B.

[37] Koelling D. D., Harmon B. N. (1977). A technique for relativistic spin-polarised calculations. J. Phys. C: Solid State Phys..

[38] Takeda T. (1978). The Scalar
Relativistic Approximation. Z. Phys. B: Condens.
Matter Quanta.

[39] Sun, L. ; Hase, W. L. Born–Oppenheimer Direct Dynamics Classical Trajectory Simulations. In Reviews in Computational Chemistry; John Wiley & Sons, Ltd., 2003; Chapter 3, pp 79–146.

[40] Nosé S. (1984). A molecular
dynamics method for simulations in the canonical ensemble. Mol. Phys..

[41] Hoover W. G. (1985). Canonical
dynamics: Equilibrium phase-space distributions. Phys. Rev. A.

[42] Chaves A. S., Piotrowski M. J., Da Silva J. L. F. (2017). Evolution of the Structural, Energetic,
and Electronic Properties of the 3d, 4d, and 5d Transition-Metal Clusters
(30 TM_n_ Systems for n= 2–15): A Density Functional
Theory Investigation. Phys. Chem. Chem. Phys..

[43] Rêgo C. R. C., Oliveira L. N., Tereshchuk P., Da Silva J. L. F. (2015). Comparative study
of van der Waals corrections to the bulk properties of graphite. J. Phys.: Condens. Matter.

[44] Rêgo C. R. C., Tereshchuk P., Oliveira L. N., Da Silva J. L. F. (2017). Graphene-supported
small transition-metal clusters: A density functional theory investigation
within van der Waals corrections. Phys. Rev.
B.

[45] Molayem M., Grigoryan V. G., Springborg M. (2011). Theoretical Determination of the
Most Stable Structures of Ni_m_Ag_n_ Bimetallic
Nanoalloys. J. Phys. Chem. C.

[46] de
Amorim R. V., Batista K. E. A., Nagurniak G. R., Orenha R. P., Parreira R. L. T., Piotrowski M. J. (2020). CO, NO,
and SO adsorption on Ni nanoclusters: a DFT investigation. Dalton Trans.

[47] Felix J. P. C. S., Batista K. E. A., Morais W. O., Nagurniak G. R., Orenha R. P., Rêgo C. R. C., Guedes-Sobrinho D., Parreira R. L. T., Ferrer M. M., Piotrowski M. J. (2023). Molecular
adsorption on coinage metal subnanoclusters: A DFT+D3 investigation. J. Comput. Chem..

[48] de
Heer W. A. (1993). The physics of simple metal clusters: experimental
aspects and simple models. Rev. Mod. Phys..

[49] Müllerová S., Malček M., Bucinsky L., Cordeiro M. N. D. S. (2024). Exploring
hydrogen binding and activation on transition metal?modified circumcoronene. Carb. Lett..

[50] Yonezawa A. F., Nagurniak G. R., Orenha R. P., da Silva E. H., Parreira R. L. T., Piotrowski M. J. (2021). Stability Changes in Iridium Nanoclusters
via Monoxide Adsorption: A DFT Study within the van der Waals Corrections. J. Phys. Chem. A.

[51] Sousa K. A. P., Morawski F. M., de Campos C. E. M., Parreira R. L. T., Piotrowski M. J., Nagurniak G. R., Jost C. L. (2022). Electrochemical, theoretical, and
analytical investigation of the phenylurea herbicide fluometuron at
a glassy carbon electrode. Electrochim. Acta.

[52] Momma K., Izumi F. (2008). VESTA: A Three-Dimensional
Visualization System for Electronic and
Structural Analysis. J. Appl. Crystallogr..

[53] Hoppe R. (1970). The Coordination
Number – an ”Inorganic Chameleon. Angew. Chem., Int. Ed..

[54] Hoppe R. (1979). Effective
Coordination Numbers (ECoN) and Mean Active Fictive Ionic Radii (MEFIR). Z. Kristallogr..

[55] Bader, R. F. W. Atoms in Molecules: A Quantum Theory; International Series of Monographs on Chemistry; Clarendon Press, 1994.

[56] Tang W., Sanville E., Henkelman G. (2009). A Grid-Based Bader Analysis Algorithm
without Lattice Bias. J. Phys.: Condens. Matter.

[57] Montoro J. C. G., Abascal J. L. F. (1993). The Voronoi Polyhedra
as Tools for
Structure Determination in Simple Disordered Systems. J. Phys. Chem..

[58] Chaves A. S., Piotrowski M. J., Guedes-Sobrinho D., Da Silva J. L. F. (2015). Theoretical Investigation
of the Adsorption Properties of CO, NO, and OH on Monometallic and
Bimetallic 13 – Atom Clusters: The Example of Cu_13_, Pt_7_Cu_6_, and Pt_13_. J. Phys. Chem. A.

[59] Tao J., Perdew J. P., Staroverov V. N., Scuseria G. E. (2003). Climbing the Density
Functional Ladder: Nonempirical Meta-Generalized Gradient Approximation
Designed for Molecules and Solids. Phys. Rev.
Lett..

[60] Lechtken A., Neiss C., Stairs J., Schooss D. (2008). Comparative study of
the structures of copper, silver, and gold icosamers: Influence of
metal type and charge state. J. Chem. Phys..

[61] Bumüller D., Yohannes A. G., Kohaut S., Kondov I., Kappes M. M., Fink K., Schooss D. (2022). Structures
of Small Platinum Cluster
Anions Ptn-: Experiment and Theory. J. Phys.
Chem. A.

[62] Kittel, C. Introduction to Solid State Physics, 7th ed.; John Wiley & Sons: New York, 1996.

[63] Pauling L. (1947). Atomic radii
and interatomic distances in metals. J. Am.
Chem. Soc..

[64] Feibelman P. J. (1996). Relaxation
of hcp(0001) surfaces: A chemical view. Phys.
Rev. B.

[65] Huber, K.-P. Molecular Spectra and Molecular Structure: IV. Constants of Diatomic Molecules; Springer Science & Business Media, 2013.

[66] Computational Chemistry Comparison and Benchmark DataBase (CCCBDB); http://cccbdb.nist.gov (accessed 2024–05–23).

[67] Irikura K. K. (2007). Experimental
Vibrational Zero-Point Energies: Diatomic Molecules. J. Phys. Chem. Ref. Data.

[68] Hammer, B. ; Nørskov, J. K. Advances in Catalysis; Academic Press Inc.: San Diego, 2000.

[69] Lamanec M., Zienertová J., Špet́ko M., Nachtigallová D., Hobza P. (2024). Similarities
and Differences of Hydridic and Protonic Hydrogen Bonding. ChemPhysChem.

[70] Hobza P., Havlas Z. (2000). Blue-Shifting Hydrogen Bonds. Chem. Rev..

[71] Belkova N.
V., Epstein L. M., Filippov O. A., Shubina E. S. (2016). Hydrogen and Dihydrogen
Bonds in the Reactions of Metal Hydrides. Chem.
Rev..

[72] Civiš S., Lamanec M., Špirko V., Kubišta J., Špet́ko M., Hobza P. (2023). Hydrogen Bonding with Hydridic Hydrogen-Experimental
Low-Temperature IR and Computational Study: Is a Revised Definition
of Hydrogen Bonding Appropriate?. J. Am. Chem.
Soc..

