# Supplementary material for: Highlighting the Potential of Synergistic Cu–Pt Single-Atom Alloy Sub-nanoclusters for Enhanced H2 Adsorption: A DFT Investigation
Source: ACS Nanosci Au. 2024 Dec 16;5(3):153–64. doi: 10.1021/acsnanoscienceau.4c00058 (PMC12183594; doi:10.1021/acsnanoscienceau.4c00058)
Supplement: Supplementary file 1 [file ng4c00058_si_001.pdf]

Supporting Information: Highlighting the Potential of Synergistic Cu – Pt Single-Atom Alloy Sub-Nanoclusters for Enhanced H_2 Adsorption: A DFT Investigation

João Paulo Cerqueira Felix,[†] Wanderson Souza Araújo,[‡] João Marcos Tomaz Palheta,[‡] Jônatas Favotto Dalmedico,[‡] Fabiano Pereira de Oliveira,[‡] Alexandre C. Dias,[¶] Diego Guedes-Sobrinho,[§] Celso R. C. Rêgo,[||] Renato L. T. Parreira,[⊥] and Maurício J. Piotrowski[*,‡]

†*Institute of Physics "Armando Dias Tavares", Rio de Janeiro State University,* 20550-900*, Rio de Janeiro, RJ, Brazil*

‡*Department of Physics, Federal University of Pelotas, PO Box* 354, 96010-900*, Pelotas, RS, Brazil*

¶*Institute of Physics and International Center of Physics, University of Brasília,* 70919 – 970*, Brasília, DF, Brazil*

§*Chemistry Department, Federal University of Paraná,* 81531 – 980*, Curitiba, PR, Brazil*

||*Institute of Nanotechnology Hermann-von-Helmholtz-Platz, Karlsruhe Institute of Technology,* 76021*, Karlsruhe, Germany*

⊥*Núcleo de Pesquisas em Ciências Exatas e Tecnológicas, Universidade de Franca,* 14404 – 600*, Franca, SP, Brazil*

E-mail: mauriciomjp@gmail.com

Contents

1 Convergence Tests **S3**

2 Sub-Nanoclusters Vibrational Frequencies **S5**

3 The Impact of Alternative Functionals (TPSS) **S6**

4 The Relativistic Effects of Spin-Orbit Coupling (SOC) **S8**

5 Sub-Nanoclusters Properties **S10**

6 *Ab Initio* Molecular Dynamics (AIMD) Simulations **S11**

7 Density Of States for Sub-Nanoclusters **S14**

8 Electronic Charge Analysis **S15**

9 Magnetic Analysis **S16**

10 Density Of States for Adsorbed Systems **S17**

11 Center of Gravity of the Occupied d States **S18**

12 H_2 Vibrational Frequencies **S19**

13 Estimation of Activation Energy **S20**

14 $H_2/Cu_{n-1}Pt$ Atomic Configuration (w/ H–H) **S22**

15 Hybridization Index **S23**

16 Atomic Coordinates **S24**

1 Convergence Tests

We conducted convergence tests to optimize the parameters for sub-nanocluster calculations, focusing on box size, cut-off energy (ENCUT), and electronic (EDIFF) and ionic (EDIFFG) convergence criteria. These tests were performed on $H_2/Cu_{11}Pt$ systems, where we analyzed various properties, including energetic stability ($\Delta E_{\text{tot}} = E_{\text{tot}}^{\text{lowest}} - E_{\text{tot}}$), structural characteristics such as effective coordination number (ECN), average bond length (d_{av}), and the H−H bond length (d_{H-H}), as well as magnetic properties (m_{tot}). The results of these analyses are presented in Tables S1 – S4.

Table S1: Box size convergence test (BOX) for the $H_2/Cu_{11}Pt$ system. This table presents the relative total energies (ΔE_{tot}), effective coordination number for the sub-nanoclusters (ECN), average bond lengths for the sub-nanoclusters (d_{av}), H−H bond distance ($d_{\text{H-H}}$), and total magnetic moments (m_{tot}). Calculations were performed using a cutoff energy of 500 eV and convergence criteria of 1.0×10^{-6} eV (energy) and 0.015 eV/Å (force).

BOX (Å)	ΔE_{tot} (eV)	ECN	d_{av} (Å)	$d_{\text{H-H}}$ (Å)	m_{tot} (μ_B)
12	0.7413	5.4696	2.4695	1.6354	1
14	0.0725	5.2099	2.4408	1.6629	1
16	0.0475	5.2119	2.4409	1.6627	1
18	0.0055	5.2118	2.4410	1.6627	1
20	0.0054	5.2118	2.4411	1.6627	1
22	0.0000	5.2117	2.4411	1.6627	1

Table S2: Cutoff energy convergence test (ENCUT) for the $H_2/Cu_{11}Pt$ system. This table presents the relative total energies (ΔE_{tot}), effective coordination number for the sub-nanoclusters (ECN), average bond lengths for the sub-nanoclusters (d_{av}), H−H bond distance ($d_{\text{H-H}}$), and total magnetic moments (m_{tot}). Calculations were performed using a cubic box with a side length of 20 Å and convergence criteria of 1.0×10^{-6} eV (energy) and 0.015 eV/Å (force).

ENCUT (eV)	ΔE_{tot} (eV)	ECN	d_{av} (Å)	$d_{\text{H-H}}$ (Å)	m_{tot} (μ_B)
200	1.3479	5.3203	2.4019	1.6374	1
300	0.0327	5.1876	2.4199	1.6619	1
400	0.0602	5.2101	2.4387	1.6627	1
450	0.0063	5.2118	2.4409	1.6627	1
500	0.0060	5.2117	2.4411	1.6627	1
600	0.0000	5.2117	2.4410	1.6627	1

Table S3: Energy criterion, electronic convergence test (EDIFF) for the $H_2/Cu_{11}Pt$ system. This table presents the relative total energies (ΔE_{tot}), effective coordination number for the sub-nanoclusters (ECN), average bond lengths for the sub-nanoclusters (d_{av}), H−H bond distance ($d_{H\text{-}H}$), and total magnetic moments (m_{tot}). Calculations were performed using a cubic box with a side length of 20 Å, a cutoff energy of 500 eV, and a force convergence criterion of 0.015 eV/Å.

EDIFF (eV)	ΔE_{tot} (eV)	ECN	d_{av} (Å)	$d_{H\text{-}H}$ (Å)	m_{tot} (μ_B)
1.0×10^{-2}	0.0398	5.2112	2.4459	1.6343	1
1.0×10^{-3}	0.0202	5.2101	2.4401	1.6625	1
1.0×10^{-4}	0.0012	5.2116	2.4409	1.6627	1
1.0×10^{-5}	0.0002	5.2117	2.4410	1.6627	1
1.0×10^{-6}	0.0001	5.2117	2.4411	1.6627	1
1.0×10^{-7}	0.0000	5.2117	2.4411	1.6627	1

Table S4: Force criterion, ionic convergence test (EDIFFG) for the $H_2/Cu_{11}Pt$ system. This table presents the relative total energies (ΔE_{tot}), effective coordination number for the sub-nanoclusters (ECN), average bond lengths for the sub-nanoclusters (d_{av}), H−H bond distance ($d_{H\text{-}H}$), and total magnetic moments (m_{tot}). Calculations were performed using a cubic box with a side length of 20 Å, a cutoff energy of 500 eV, and an energy convergence criterion of 1.0×10^{-6} eV.

EDIFFG (eV/Å)	ΔE_{tot} (eV)	ECN	d_{av} (Å)	$d_{H\text{-}H}$ (Å)	m_{tot} (μ_B)
0.0500	0.0067	5.2110	2.4405	1.6338	1
0.0100	0.0013	5.2115	2.4410	1.6627	1
0.0050	0.0009	5.2116	2.4411	1.6627	1
0.0025	0.0001	5.2116	2.4410	1.6627	1
0.0010	0.0000	5.2117	2.4410	1.6627	1
0.0001	0.0000	5.2117	2.4411	1.6627	1

2 Sub-Nanoclusters Vibrational Frequencies

Figure S1 presents the vibrational frequencies (ν) for the lowest energy configurations of Cu_n ($n = 2-14$) sub-nanoclusters, as well as for the lowest energy sizes of Cu_n and $Cu_{n-1}Pt$ (n = 4, 6, 8, 10, and 12). For linear configurations, the $3n-5$ vibrational modes are shown, while for non-linear configurations, the $3n-6$ vibrational modes are depicted.

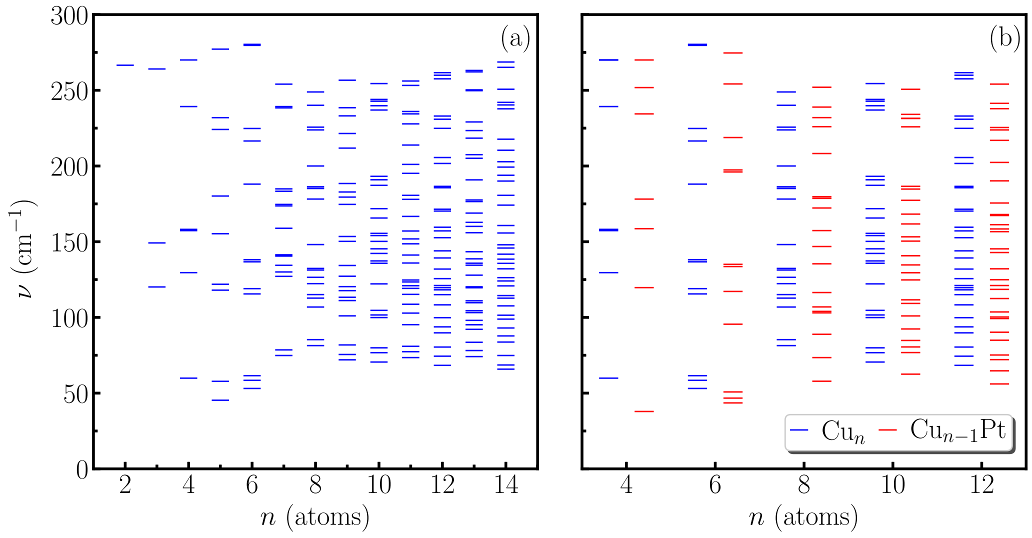

Figure S1: (a) Vibrational frequencies (ν, in cm^{-1}) for the lowest energy configurations of Cu_n ($n = 2-14$) sub-nanoclusters. (b) Vibrational frequencies for the lowest energy sizes of Cu_n (shown in blue) and $Cu_{n-1}Pt$ (shown in red) for n = 4, 6, 8, 10, and 12.

3 The Impact of Alternative Functionals (TPSS)

In Figure S2, we present the most stable Cu_n sub-nanoclusters and their corresponding $Cu_{n-1}Pt$ sub-nanoclusters ($n = 4$, 6, 8, 10, and 12), obtained using the PBE+D3 and TPSS+D3 protocols.

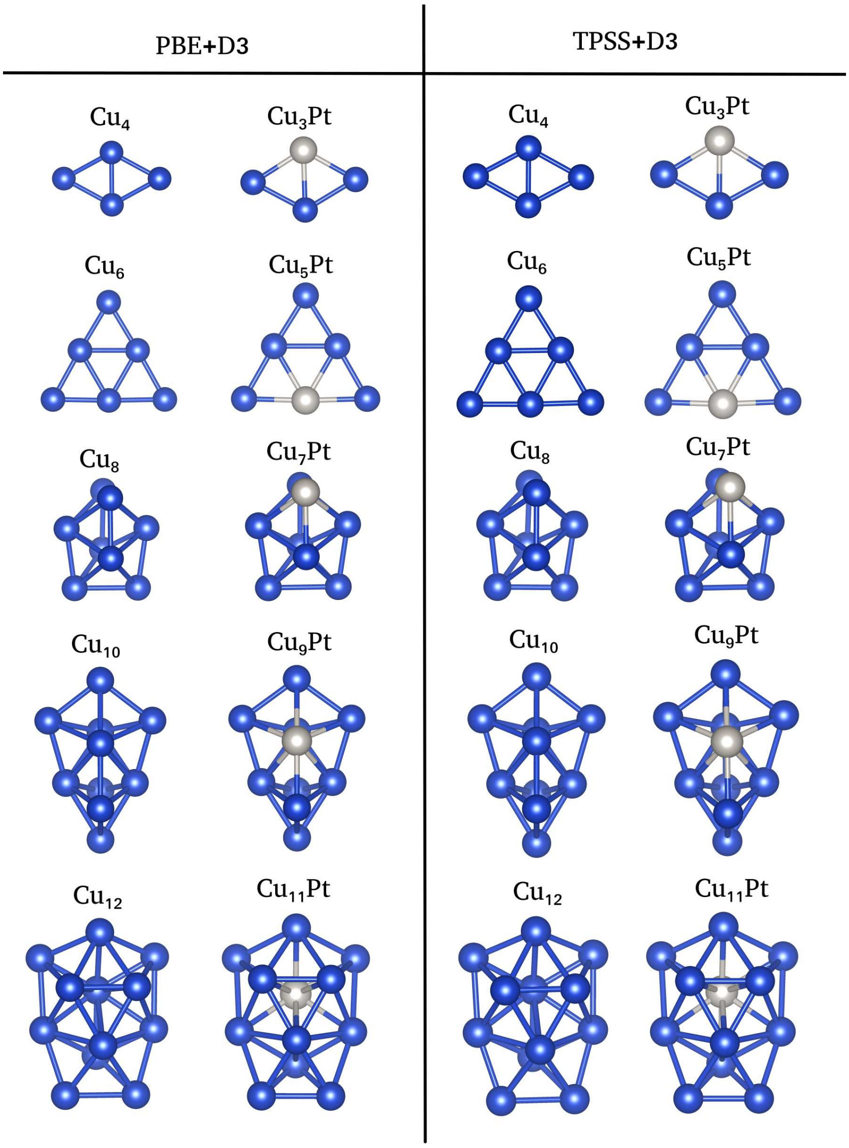

Figure S2: The most stable sizes of Cu_n sub-nanoclusters and their corresponding most stable $Cu_{n-1}Pt$ sub-nanoclusters ($n = 4$, 6, 8, 10, and 12), as determined using the PBE+D3 and TPSS+D3 protocols.

In Table S5, we present a comparison of properties for the PBE+D3 and TPSS+D3 protocols applied to Cu_n and $Cu_{n-1}Pt$ sub-nanoclusters (n = 4, 6, 8, 10, and 12). The properties include binding energy (E_b), average bond length (d_{av}), and effective coordination number (ECN), along with the corresponding percentage differences between the two protocols.

Table S5: Comparison of properties between the PBE+D3 and TPSS+D3 protocols, including binding energy (E_b), average bond length (d_{av}), and effective coordination number (ECN), along with the corresponding percentage differences between the two protocols for Cu_n and $Cu_{n-1}Pt$ sub-nanoclusters (n = 4, 6, 8, 10, and 12).

	PBE+D3			TPSS+D3			Diff.		
System	E_b (eV/atom)	d_{av} (Å)	ECN	E_b (eV/atom)	d_{av} (Å)	ECN	ΔE_b (%)	Δd_{av} (%)	ΔECN (%)
Cu_4	-1.6057	2.3486	2.4767	-1.6239	2.3486	2.4767	-1.13	0.00	0.00
Cu_6	-1.9450	2.3490	2.9859	-1.9738	2.3490	2.9859	-1.48	0.00	0.00
Cu_8	-2.1838	2.4206	4.4969	-2.2609	2.4206	4.4969	-3.52	0.00	0.00
Cu_{10}	-2.2687	2.4375	5.1513	-2.3612	2.4098	5.1599	-4.07	1.13	-0.16
Cu_{12}	-2.3638	2.4473	5.5319	-2.4688	2.4473	5.5319	-4.44	0.00	0.00
Cu_3Pt	-1.8808	2.3930	2.4973	-1.9041	2.3768	2.4927	-1.23	0.67	0.18
Cu_5Pt	-2.1254	2.3854	2.9709	-2.1576	2.3659	2.9704	-1.51	0.81	0.01
Cu_7Pt	-2.3563	2.4436	4.5128	-2.4355	2.4185	4.5251	-3.36	1.02	-0.27
Cu_9Pt	-2.4385	2.4680	5.1437	-2.5281	2.4410	5.1419	-3.67	1.09	0.03
$Cu_{11}Pt$	-2.5072	2.4825	5.4869	-2.6082	2.4553	5.4924	-4.02	1.09	-0.10

4 The Relativistic Effects of Spin-Orbit Coupling (SOC)

In Figure S3, we present the most stable Cu_n sub-nanoclusters and their corresponding $Cu_{n-1}Pt$ sub-nanoclusters ($n = 4, 6, 8, 10$, and 12), as obtained from the PBE+D3 and PBE+D3+SOC protocols.

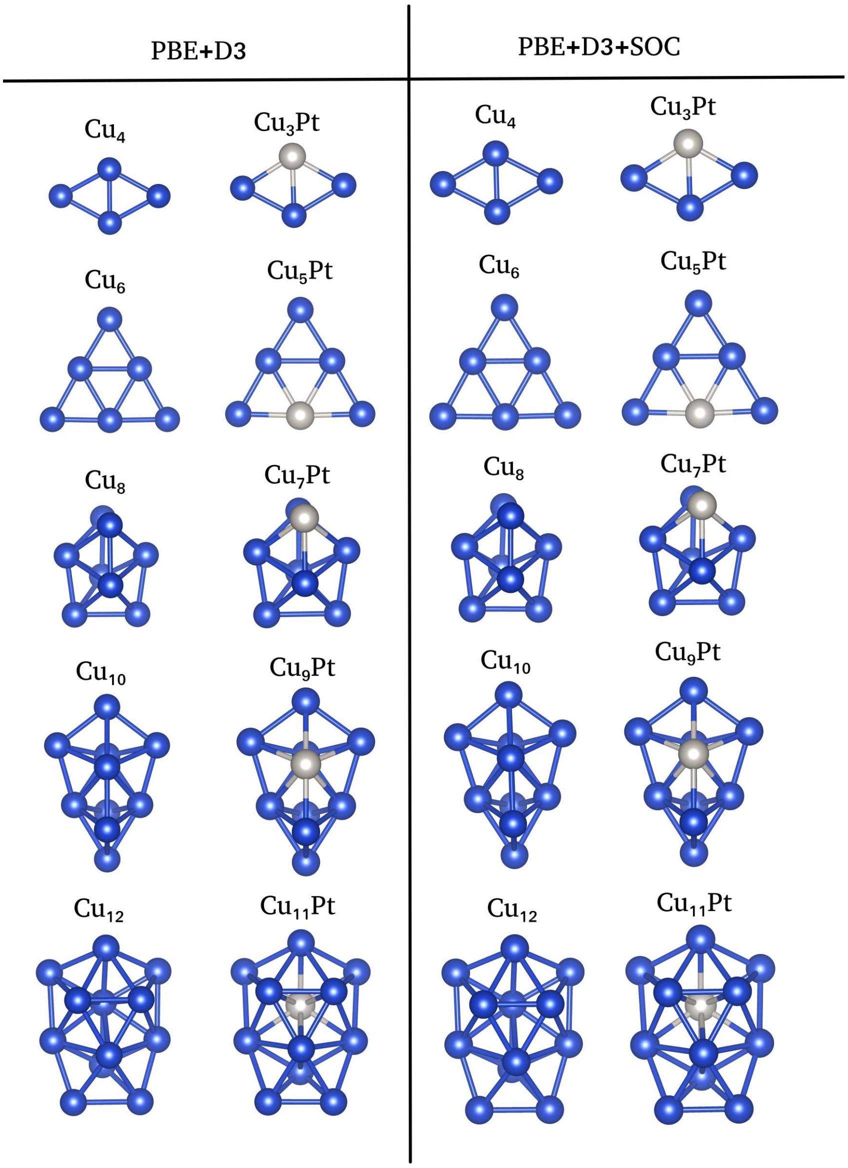

Figure S3: The most stable sizes for Cu_n sub-nanoclusters and the corresponding $Cu_{n-1}Pt$ sub-nanoclusters ($n = 4, 6, 8, 10$, and 12) calculated using the PBE+D3 and PBE+D3+SOC protocols.

In Table S6, we present a comparison of properties for the PBE+D3 and PBE+D3+SOC protocols applied to Cu_n and $Cu_{n-1}Pt$ sub-nanoclusters (n = 4, 6, 8, 10, and 12). The properties compared include binding energy (E_b), average bond length (d_{av}), and effective coordination number (ECN), along with the corresponding percentage differences between the two protocols.

Table S6: Comparison of properties between the PBE+D3 and PBE+D3+SOC protocols, including binding energy (E_b), average bond length (d_{av}), and effective coordination number (ECN), along with the corresponding percentage differences between the two protocols for Cu_n and $Cu_{n-1}Pt$ sub-nanoclusters (n = 4, 6, 8, 10, and 12).

	PBE+D3			PBE+D3+SOC			Diff.		
System	E_b (eV/atom)	d_{av} (Å)	ECN	E_b (eV/atom)	d_{av} (Å)	ECN	ΔE_b (%)	Δd_{av} (%)	ΔECN (%)
Cu_4	-1.6057	2.3486	2.4767	-1.6239	2.3486	2.4767	-1.13	0.00	0.00
Cu_6	-1.9450	2.3490	2.9859	-1.9738	2.3490	2.9859	-1.48	0.00	0.00
Cu_8	-2.1838	2.4206	4.4969	-2.2609	2.4206	4.4969	-3.52	0.00	0.00
Cu_{10}	-2.2687	2.4375	5.1513	-2.3612	2.4098	5.1599	-4.07	1.13	-0.16
Cu_{12}	-2.3638	2.4473	5.5319	-2.4688	2.4473	5.5319	-4.44	0.00	0.00
Cu_3Pt	-1.8808	2.3930	2.4973	-1.9041	2.3768	2.4927	-1.23	0.67	0.18
Cu_5Pt	-2.1254	2.3854	2.9709	-2.1576	2.3659	2.9704	-1.51	0.81	0.01
Cu_7Pt	-2.3563	2.4436	4.5128	-2.4355	2.4185	4.5251	-3.36	1.02	-0.27
Cu_9Pt	-2.4385	2.4680	5.1437	-2.5281	2.4410	5.1419	-3.67	1.09	0.03
$Cu_{11}Pt$	-2.5072	2.4825	5.4869	-2.6082	2.4553	5.4924	-4.02	1.09	-0.10

5 Sub-Nanoclusters Properties

In Figure S4, we present the binding energy (E_b), average bond length (d_{av}), effective coordination number (ECN), total magnetic moment (m_{tot}), and stability function ($\Delta^2 E$) plotted against the number of atoms, n, for the lowest energy configurations of Cu_n ($n = 2 - 14$) sub-nanoclusters.

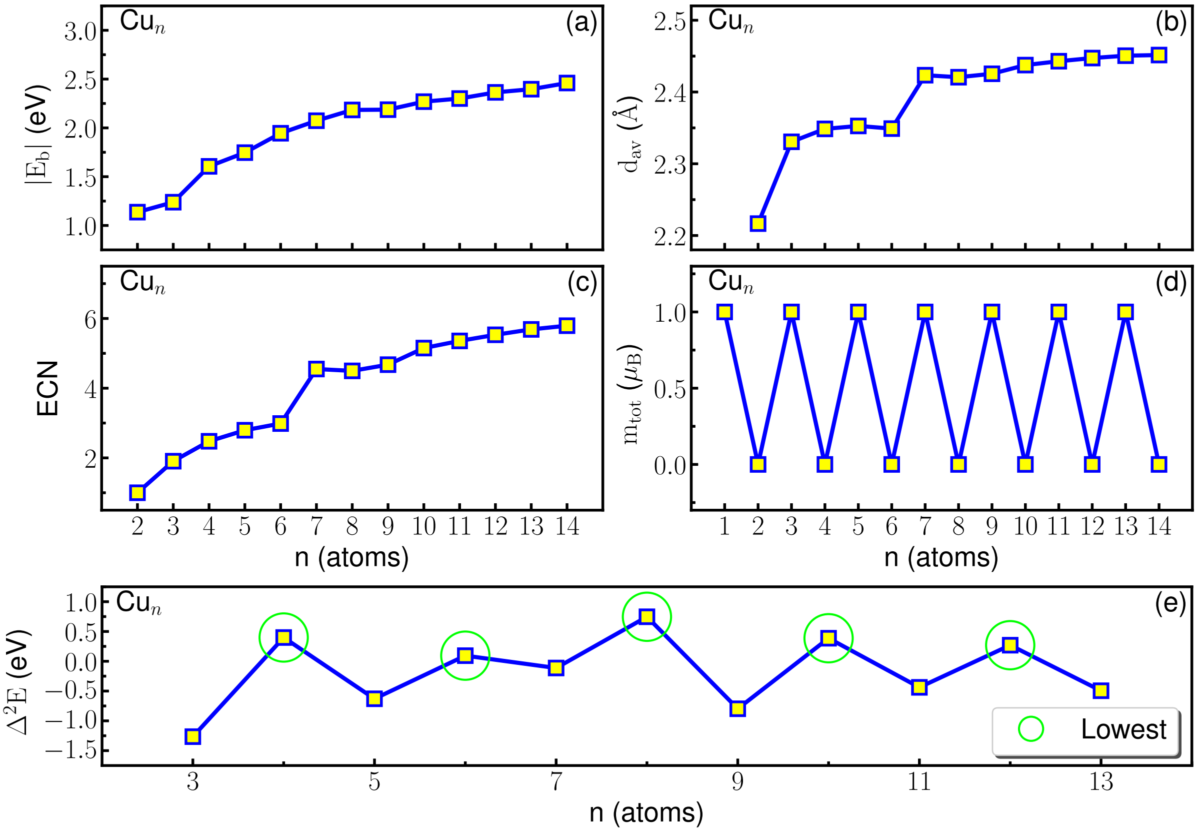

Figure S4: Properties of the lowest energy configurations of Cu_n ($n = 2-14$) sub-nanoclusters: (a) magnitude of the binding energy ($|E_b|$), (b) average bond length (d_{av}), (c) effective coordination number (ECN), (d) total magnetic moment (m_{tot}), (e) stability function ($\Delta^2 E$). All properties are presented as a function of the number of atoms, n.

6 *Ab Initio* Molecular Dynamics (AIMD) Simulations

In Figure S5, we present the AIMD simulations showing the thermalization process at 300 K for the Cu_n and $Cu_{n-1}Pt$ sub-nanoclusters ($n = 4$, 6, 8, 10, and 12). The results confirm the thermodynamic stability, as the same structural motif is maintained throughout the simulations and in the final structural optimizations.

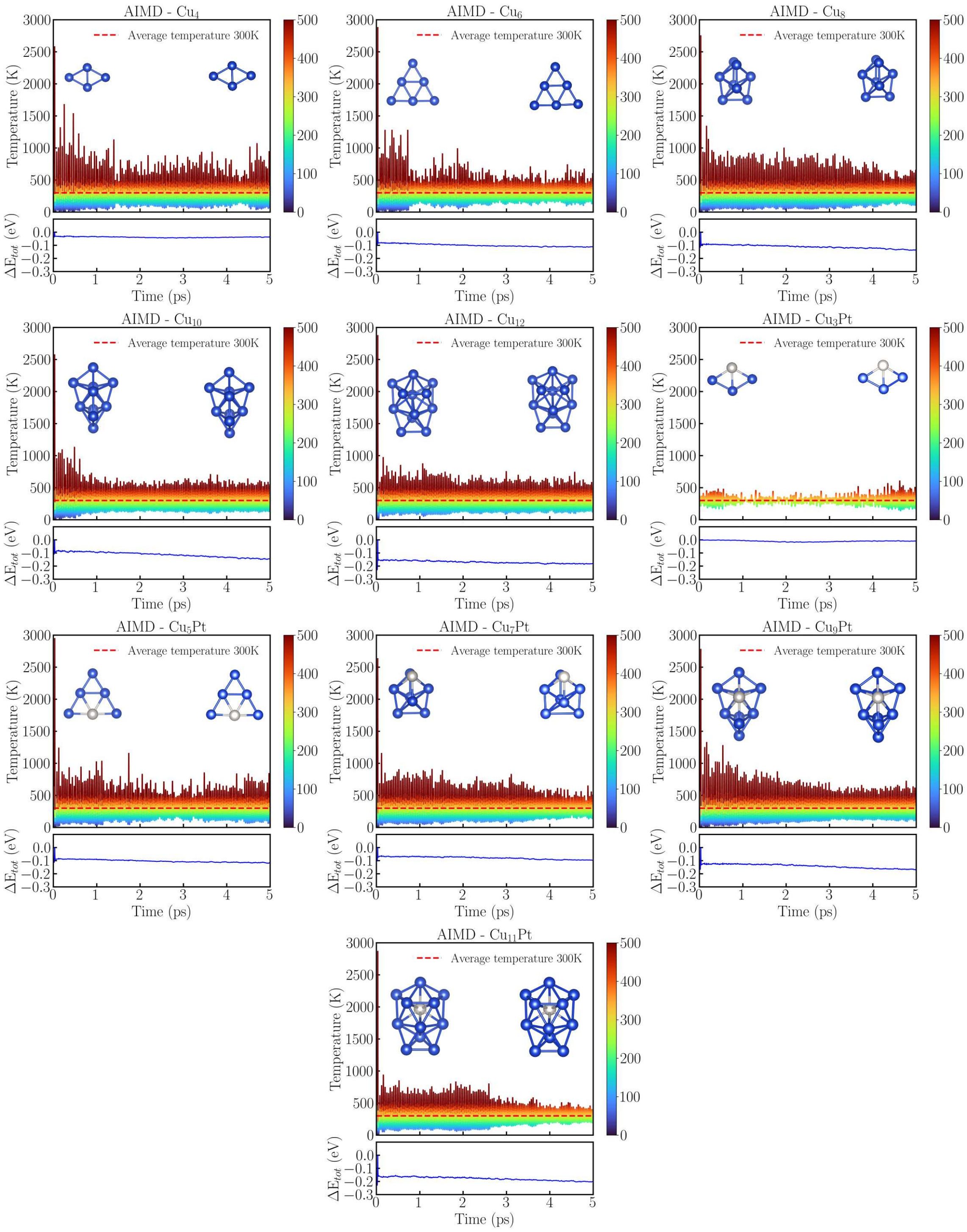

Figure S5: AIMD thermalization process at 300 K, illustrating the temperature and relative total energy as functions of the simulation steps for Cu_n and $Cu_{n-1}Pt$ sub-nanoclusters ($n = 4, 6, 8, 10, 12$). For each sub-nanocluster, the initial structure (corresponding to the tested configuration) and the final structure (last AIMD snapshot, followed by optimization) are shown.

In Table S7, we present the E_b, d_{av}, and ECN properties for the initial and final Cu_n and

$Cu_{n-1}Pt$ sub-nanocluster configurations from Figure S5, confirming that the main properties of these structures remain consistent throughout the AIMD simulations.

Table S7: Comparison of selected properties (E_b, d_{av}, and ECN) for Cu_n and $Cu_{n-1}Pt$ sub-nanoclusters ($n = 4, 6, 8, 10, 12$), before (initial structure corresponding to the tested configuration) and after (final structure obtained from the last AIMD snapshot followed by optimization) the AIMD simulations.

Before	E_b (eV)	d_{av} (Å)	ECN
Cu_4	−1.6058	2.3486	2.4769
Cu_6	−1.9450	2.3491	2.9853
Cu_8	−2.1839	2.4207	4.4961
Cu_{10}	−2.2687	2.4375	5.1529
Cu_{12}	−2.3638	2.4473	5.5325
Cu_3Pt	−1.8811	2.3980	2.4978
Cu_5Pt	−2.1254	2.3856	2.9717
Cu_7Pt	−2.3553	2.4358	4.4637
Cu_9Pt	−2.4384	2.4683	5.1437
$Cu_{11}Pt$	−2.5072	2.4824	5.4868

After	E_b (eV)	d_{av} (Å)	ECN
Cu_4	−1.6058	2.3486	2.4767
Cu_6	−1.9450	2.3490	2.9859
Cu_8	−2.1839	2.4206	4.4969
Cu_{10}	−2.2688	2.4375	5.1513
Cu_{12}	−2.3638	2.4473	5.5319
Cu_3Pt	−1.8808	2.3930	2.4973
Cu_5Pt	−2.1255	2.3854	2.9709
Cu_7Pt	−2.3563	2.4436	4.5128
Cu_9Pt	−2.4385	2.4680	5.1437
$Cu_{11}Pt$	−2.5072	2.4825	5.4869

7 Density Of States for Sub-Nanoclusters

In Figure S6, we present the local density of states (LDOS) for the lowest energy Cu_n and $Cu_{n-1}Pt$ sub-nanoclusters. The figure showcases the total and partial contributions (*s*-, *p*-, and *d*-states) for both the Cu and CuPt systems. To enhance visualization, we applied broadening using a Gaussian function, ensuring it does not affect the general conclusions drawn from the data.

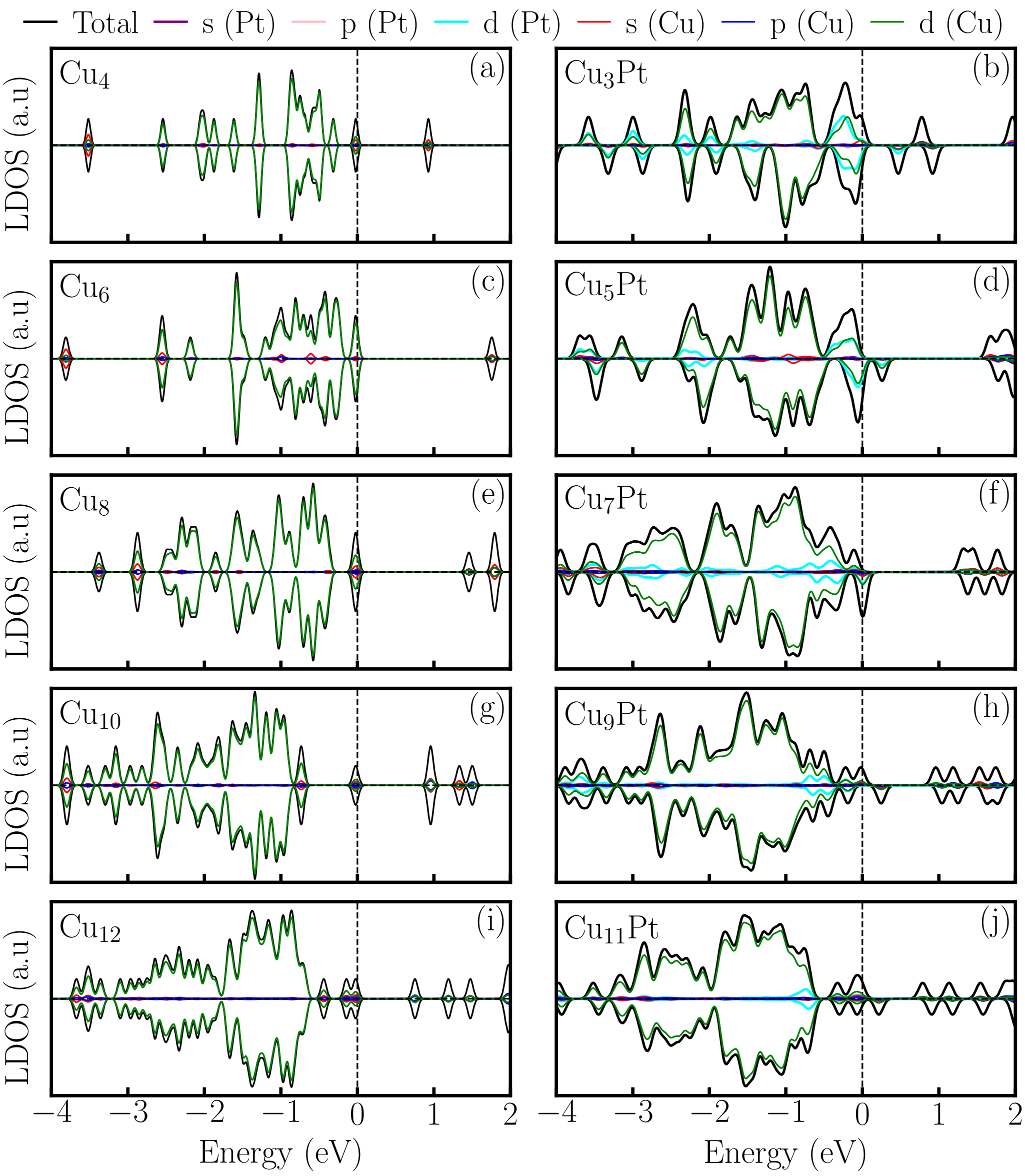

Figure S6: Local density of states (LDOS) for Cu_n and $Cu_{n-1}Pt$ sub-nanoclusters, where $n =$ 4, 6, 8, 10, and 12. The figure displays the total DOS in black color and decomposed DOS in red (purple), blue (pink), and green (cyan) colors for *s*, *p*, and *d*-states, respectively, of the Cu (Pt) atoms.

8 Electronic Charge Analysis

In Figure S7, we present the charge density for the Cu_n and $Cu_{n-1}Pt$ sub-nanoclusters. Additionally, Figure S8 displays the difference of charge density for the $Cu_{n-1}Pt$ systems. The charge density difference, $\Delta\rho$, quantifies the redistribution of electronic charge due to the substitution of a Cu atom with a Pt atom in the $Cu_{n-1}Pt$ system, relative to the corresponding Cu_n system.

$$\Delta\rho = \rho^{\mathrm{Cu}_{n-1}\mathrm{Pt}} - \rho^{\mathrm{Cu}_n} . \tag{1}$$

Here, $\rho^{\mathrm{Cu}_{n-1}\mathrm{Pt}}$ and ρ^{Cu_n} represent the charge densities of the $Cu_{n-1}Pt$ and Cu_n systems, respectively.

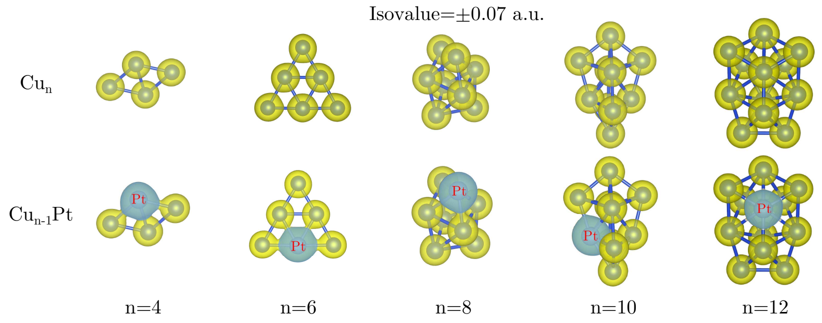

Figure S7: Charge density for Cu_n and $Cu_{n-1}Pt$ sub-nanoclusters, where $n = 4$, 6, 8, 10, and 12. The isosurface is depicted in yellow for Cu atoms and transparent blue for Pt atoms, with Pt atoms highlighted in red.

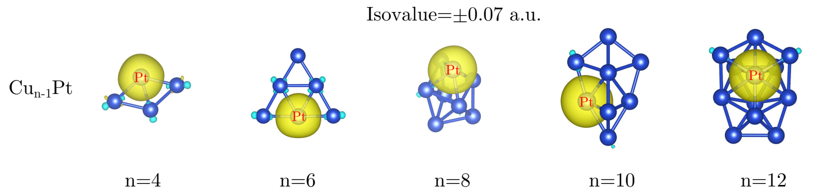

Figure S8: Difference of charge density for the $Cu_{n-1}Pt$ sub-nanoclusters, where $n = 4$, 6, 8, 10, and 12. Accumulation regions are depicted in yellow, while depletion regions are shown in cyan. Pt atoms are highlighted in red.

9 Magnetic Analysis

In Figure S9, we present the magnetization density for the $Cu_{n-1}Pt$ systems.

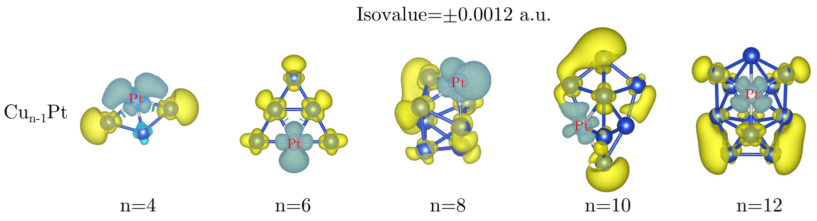

Figure S9: Magnetization density of the $Cu_{n-1}Pt$ sub-nanoclusters, where $n = 4, 6, 8, 10$, and 12. The isosurface is depicted in yellow for Cu atoms and transparent blue for Pt atoms, with Pt atoms highlighted in red.

10 Density Of States for Adsorbed Systems

In Figure S10, we present the local density of states (LDOS) for the lowest energy H_2/Cu_n and $H_2/Cu_{n-1}Pt$ systems. The figure illustrates the total and partial (*s*-, *p*-, and *d*-states) contributions for H_2 adsorbed on both Cu and CuPt systems. To enhance visualization, we applied broadening using a Gaussian function, which does not affect the general conclusions drawn from the data.

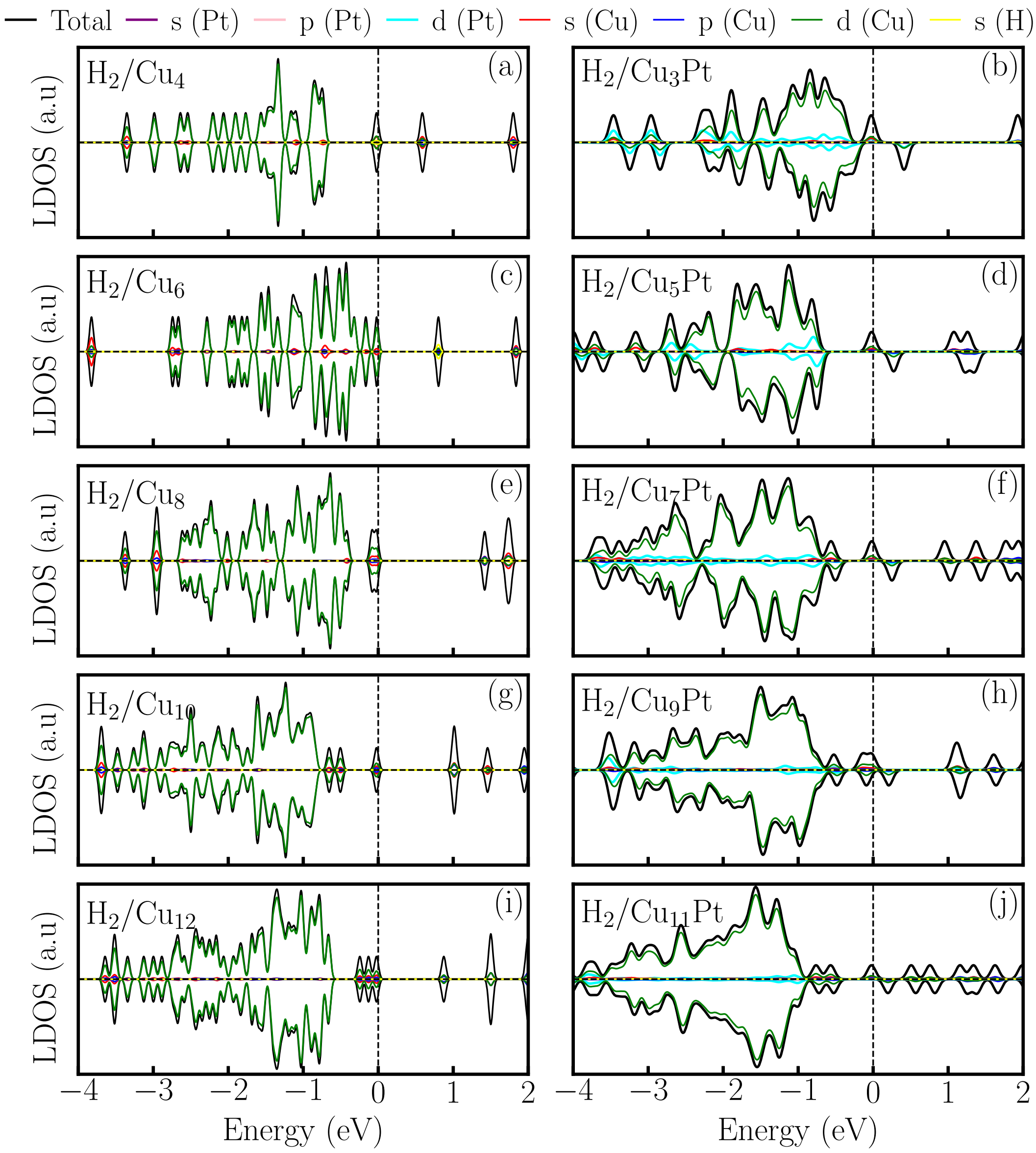

Figure S10: Local density of states (LDOS) for H_2/Cu_n and $H_2/Cu_{n-1}Pt$ systems, where $n = 4$, 6, 8, 10, and 12. The total DOS is depicted in black, while the decomposed DOS is shown in red (purple), blue (pink), and green (cyan) colors for *s*, *p*, and *d*-states, respectively, of the Cu (Pt) atoms.

11 Center of Gravity of the Occupied d States

In Figure S11, the center of gravity of the occupied d states (ε_d) is depicted for the Cu_n and $Cu_{n-1}Pt$ sub-nanoclusters, as well as for the H_2/Cu_n and $H_2/Cu_{n-1}Pt$ systems, as a function of n (4, 6, 8, 10, and 12).

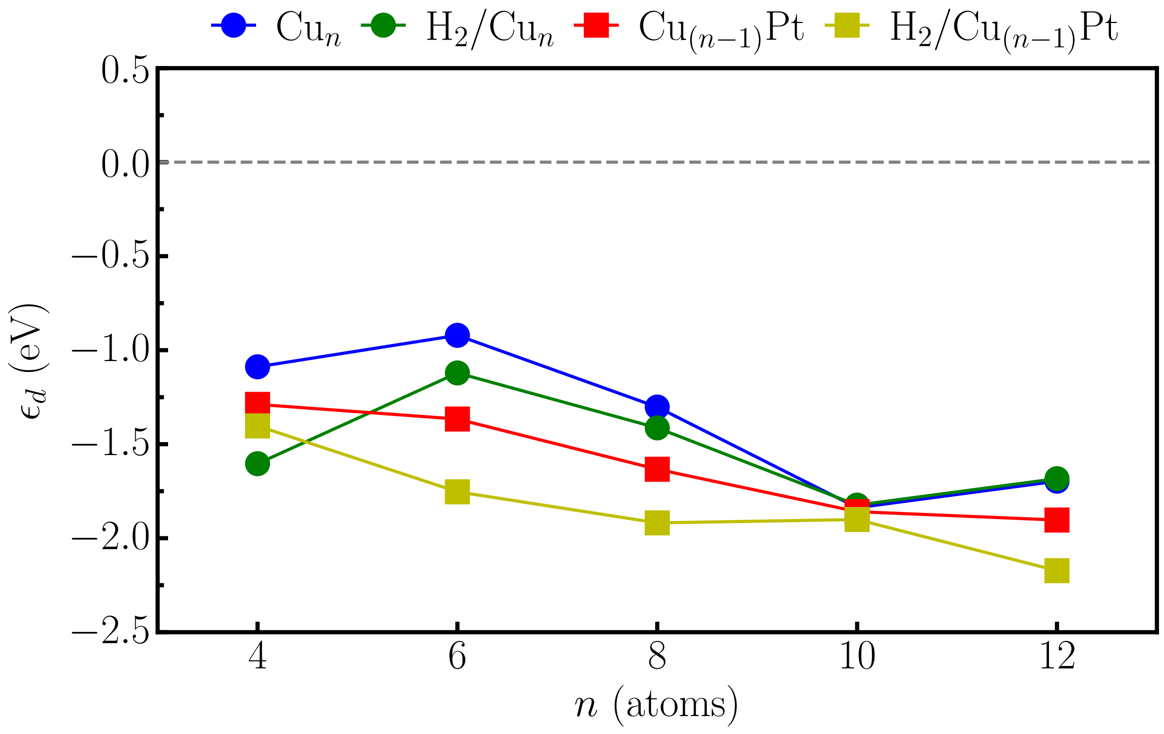

Figure S11: Center of gravity of the occupied d states (ε_d) for the Cu_n, H_2/Cu_n, $Cu_{n-1}Pt$, and $H_2/Cu_{n-1}Pt$ systems, where $n = 4$, 6, 8, 10, and 12.

12 H_2 Vibrational Frequencies

Figure S12 presents the vibrational frequencies (ν) for the H_2 molecule adsorbed on the lowest energy configurations in the H_2/Cu_n and $H_2/Cu_{n-1}Pt$ systems, as a function of n ($n =$ 4, 6, 8, 10, and 12). For reference, the vibrational frequency of H_2 in the gas phase is also presented.

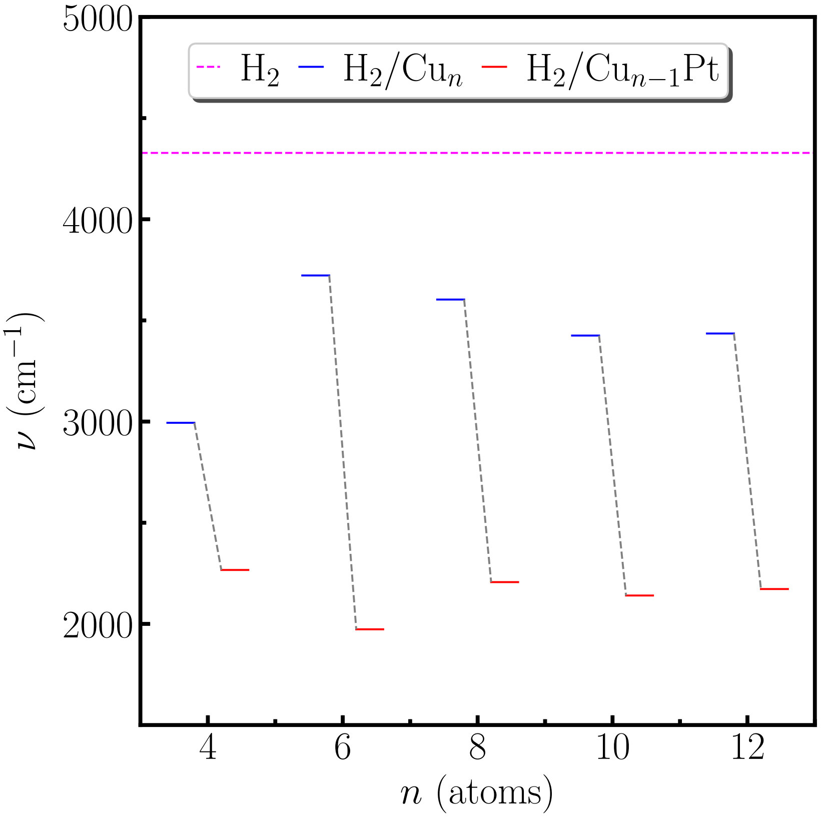

Figure S12: Vibrational frequencies (ν, in cm^{-1}) for the H_2 molecule in the lowest energy configurations of H_2/Cu_n and $H_2/Cu_{n-1}Pt$ systems as a function of n (4, 6, 8, 10, and 12). The vibrational frequency of H_2 in the gas phase is also indicated for reference.

13 Estimation of Activation Energy

Figure S13 presents a possible reaction path for H_2 dissociation, showing the initial (precursor), transition, and final states, given by the numbers 1, 2, and 3, respectively, along with the corresponding activation (E_a) and reaction (ΔE_r) energy values. The activation energy E_a was calculated as the difference between the total energies of the transition state ($E_{\text{tot},2}$) and the initial state ($E_{\text{tot},1}$):

$$E_a = E_{\text{tot},2} - E_{\text{tot},1} \; . \tag{2}$$

The reaction energy (ΔE_r) was determined as the energy difference between the final ($E_{\text{tot},3}$) and initial ($E_{\text{tot},1}$) states:

$$\Delta E_r = E_{\text{tot},3} - E_{\text{tot},1} \; . \tag{3}$$

The 'initial state' configurations (1) represent the precursor states, where H_2 is bound to the sub-nanocluster with the H–H bond intact. The 'final state' configurations (3) correspond to the co-adsorbed dissociated products, where the H–H bond is broken. The 'transition state' configurations (2) depict the intermediate mechanism for H_2 activation, transitioning from the precursor state via a top-bound pathway.

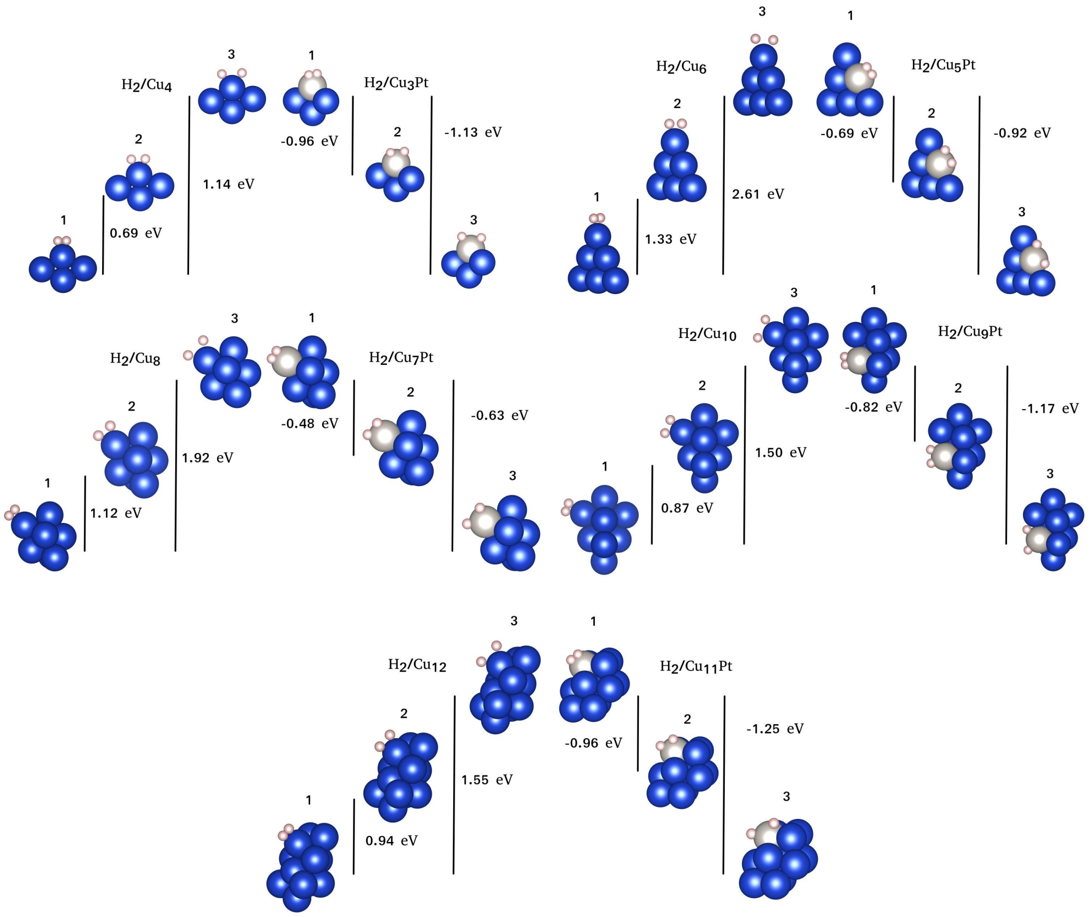

Figure S13: The schematic representation illustrates the initial (1), transition (2), and final (3) states for the most stable H_2/Cu_n and $H_2/Cu_{n-1}Pt$ systems ($n = 4$, 6, 8, 10, and 12). It includes the activation energy (between states 1 and 2) and the reaction energy (between states 1 and 3). State 2 represents the transition state, modelled as a top-bound configuration.

14 $H_2/Cu_{n-1}Pt$ Atomic Configuration (w/ H – H)

Figure S14 presents the atomic configurations for $H_2/Cu_{n-1}Pt$ sub-nanoclusters, considering the most stable higher-energy $H_2/Cu_{n-1}Pt$ systems where the H – H bond is intact (w/ H – H), along with the corresponding relative total energies compared to the lowest-energy $H_2/Cu_{n-1}Pt$ configurations (w/o H – H).

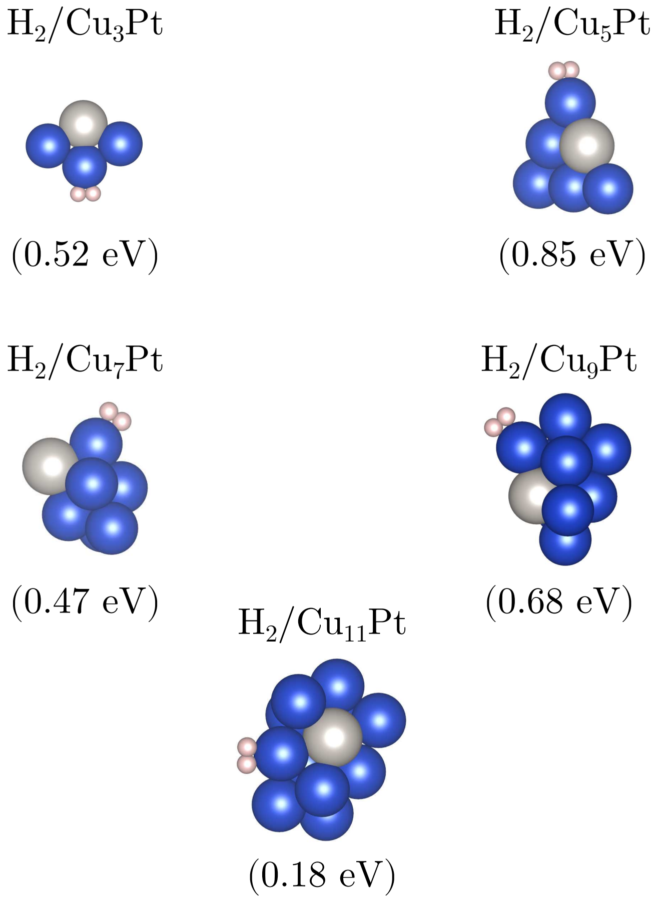

Figure S14: The atomic configurations for $H_2/Cu_{n-1}Pt$ sub-nanoclusters, with $n = 4$, 6, 8, 10, and 12, focusing on the most stable higher-energy $H_2/Cu_{n-1}Pt$ systems where the H – H bond is present (w/ H – H). Below each configuration, the relative total energy is provided in comparison to the lowest-energy $H_2/Cu_{n-1}Pt$ systems, where the H – H bond is absent (w/o H – H).

15 Hybridization Index

In Table S8, the *sd*, *sp*, and *pd* hybridization indexes are presented for both up and down spin cases in the H_2/Cu_n and $H_2/Cu_{n-1}Pt$ systems.

Table S8: Hybridization index for *sd*, *sp*, and *pd* cases, considering both up and down spin states, for the H_2/Cu_n and $H_2/Cu_{n-1}Pt$ systems, where $n = 4$, 6, 8, 10, and 12.

Hybrid.	H_2/Cu_4	H_2/Cu_3Pt	H_2/Cu_6	H_2/Cu_5Pt	H_2/Cu_8	H_2/Cu_7Pt	H_2/Cu_{10}	H_2/Cu_9Pt	H_2/Cu_{12}	$H_2/Cu_{11}Pt$
sd up	0.0793	0.0947	0.0903	0.0823	0.0502	0.0628	0.0561	0.0636	0.0554	0.0577
sp up	0.0082	0.0042	0.0071	0.0041	0.0099	0.0058	0.0105	0.0060	0.0116	0.0068
pd up	0.0178	0.0286	0.0232	0.0239	0.0315	0.0355	0.0352	0.0398	0.0343	0.0396
sd down	0.0793	0.0888	0.0903	0.0866	0.0502	0.0636	0.0561	0.0653	0.0554	0.0572
sp down	0.0082	0.0037	0.0071	0.0035	0.0099	0.0047	0.0105	0.0057	0.0116	0.0063
pd down	0.0178	0.0265	0.0232	0.0222	0.0315	0.0343	0.0352	0.0378	0.0343	0.0385

16 Atomic Coordinates

Below, we provided the XYZ coordinates of the lowest energy Cu_n and $Cu_{n-1}Pt$ sub-nanoclusters.

Cu_n

Cu_2

2

Cu 0.0000000000000000 0.0000000000000000 -1.1082991050001212

Cu 0.0000000000000000 0.0000000000000000 1.1082991050001212

Cu_3

3

Cu -1.0163713573783593 -0.7651048298696388 0.0000000000000000

Cu -0.2420379904215206 1.3796160051375654 0.0000000000000000

Cu 1.2584093477998817 -0.6145111752679266 0.0000000000000000

Cu_4

4

Cu -0.3033580792804820 0.9851112112234901 0.4672786574856662

Cu 0.3032454706228620 -0.9869540462005215 -0.4637031793688102

Cu 2.0043414255248688 0.4748185620496344 0.3010859658445302

Cu -2.0042288168672489 -0.4729757270726047 -0.3046614439613844

Cu_5

5

Cu -0.3936207908312443 1.6685336908775561 0.0000000000000000

Cu 1.6509538689350265 0.4592777629121905 0.0000000000000000

Cu -2.4182335731920475 0.4777024562286236 0.000000000000000

Cu -0.4232847458275621 -0.7161699221240614 0.0000000000000000

Cu 1.5841852409158328 -1.8893439878943141 0.0000000000000000

Cu_6

6

Cu -1.2691141651789977 -0.2189092236331103 0.5147754367734105

Cu 2.4631109559183209 0.4284419524237357 -1.0037766985187737

Cu -1.5074704080182215 -2.0213324206078980 -0.9473095888677410

Cu 0.4923648903862681 -0.8206189570137958 -1.0042560408349726

Cu -0.9532780251239696 1.5895381074889237 1.9548372431111964

Cu 0.7743867520165999 1.0428805413421429 0.4857296483368803

Cu_7

7

Cu -1.2317361540795950 -1.6458653234819760 -0.0233983298213563

Cu 0.6913974249830694 -1.3309468420272950 1.4065758045578747

Cu 0.3360318906799371 1.8381181147450913 -0.8571340711959969

Cu 0.7480224817844068 -0.5449311146770697 -0.8792597238874489

Cu 1.6528088840852853 0.8283328252648037 0.8997477185601817

Cu -0.7481806395603989 0.5444937524191005 0.8793954628735694

Cu -1.4483438878927029 0.3107985877573505 -1.4259268610868219

Cu_8

8

Cu 1.1397218164636325 1.9007007973183008 0.2156149240453900

Cu 1.5435618210136646 -0.4754979155410197 0.3668316362310531

Cu -2.1311998711092706 -0.5501682270815813 0.3392554494022413

Cu -0.9038687497383258 1.1259205245468458 -0.8108869227085105

Cu -0.2913172921274754 -1.2314086469849208 -1.0678987959288424

Cu -0.1930433860631240 -1.8173042107018933 1.2725054602062578

Cu -0.3484608353315224 0.5800259735055100 1.5109096702897595
Cu 1.1846064968924210 0.4677317049387639 -1.8263314215373541

Cu_9

9

Cu -0.2481419450993805 -1.0485141031352381 -1.6779558731137243
Cu 1.8852722749162112 -0.1867234434780585 -2.3598697734716900
Cu 1.3695876488647052 -0.3225227837907916 -0.0078242649355680
Cu -1.6840684950813838 0.1751020471134179 2.0198550506149378
Cu 0.3237971612956407 1.3710020515974968 -1.4122454363943469
Cu -1.6846693977340212 0.4415571804224605 -0.3934974140951724
Cu 0.0367215574054605 1.5131257501767266 0.9504258340347267
Cu 0.6837579220757632 -0.4098367801624896 2.2659144075291451
Cu -0.6822567266429758 -1.5331899187435241 0.6151974698316920

Cu_{10}

10

Cu -0.8544945001758091 -0.0010573415955939 1.2718128515914078
Cu -0.8544517003070542 -0.0011816376304168 -1.2718329677682565
Cu -1.4837673805169072 1.9257425022114116 -0.0001345009834370
Cu 0.8563862094549695 -1.2706850514716699 0.0001530340382860
Cu 0.8526667070431060 1.2729277893610131 -0.0001295831164168
Cu -2.9851007759804356 -0.0044056471241412 -0.0000563501233071
Cu -1.4786100690517525 -1.9298626122987201 0.0000432277806990
Cu 1.4810939230321978 0.0021374371426663 1.9278604506602885
Cu 1.4811896443035817 0.0018706893096176 -1.9277773968904306
Cu 2.9850879421981062 0.0045138720958455 0.0000612348111630

Cu_{11}

11

Cu -0.4299884757969945 -0.3539628805957733 -1.7608247422271130

Cu 0.8705112247751963 -1.0457730069639641 0.2571188950399517

Cu -0.5445800072460560 -0.2857089351146431 2.1518276842661628

Cu -2.6693600137613771 0.4015067791808686 -1.0340810569404351

Cu 1.9577061690356459 -0.9854145662356810 -1.8866266805096430

Cu -2.7757502397670102 0.4007323978433792 1.3865923004649954

Cu 1.4158303771238678 0.9988226432569487 1.4161457093811354

Cu -1.5228562198326117 -1.3025742808145413 0.1972065963153860

Cu 1.3273455269246597 1.1445035422405496 -0.9902576631326330

Cu 3.1255789963944061 -0.1118081100505535 0.0460534406048456

Cu -0.7544373378497440 1.1396764172534173 0.2168455167373633

Cu_{12}

12

Cu -0.6413470418507732 -0.2783309728008589 -1.9691156232979221

Cu 0.6770476887374794 -0.9700495034118521 -0.0036765522710720

Cu -0.6183656651460030 -0.2790322964085501 1.9762390867882704

Cu -2.8774982873815684 0.4704816507477236 -1.2469391908025038

Cu 1.8566972563463082 -0.8889950260997566 -2.0652814804226143

Cu -2.8631770075491101 0.4702043893679733 1.2795127626682063

Cu 1.1773743592904857 1.2186007102694045 1.1914037397476243

Cu -1.7298773834179819 -1.1846915862233445 0.0097124071274397

Cu 1.8816728010608674 -0.8894592936447214 2.0434778363038628

Cu 1.1635122652615042 1.2188562452236642 -1.2041141349051276

Cu 2.9227733794481079 -0.0904912416696515 -0.0167297863177360

Cu -0.9488123647993003 1.2029069246499606 0.0055109353815705

Cu_{13}

13

Cu	-0.8010018397858545	-2.4229840607083020	-1.3866246921414191
Cu	-2.7190097493147238	0.1348548848213689	1.0109978248606399
Cu	0.2334093906577337	-0.7745864515260532	-2.8761857475361428
Cu	0.8638795328412989	1.8598740614859857	2.3979007913964328
Cu	1.0525758158820331	-0.4388395064577502	1.7957497568623619
Cu	-1.0381633027844153	0.2523913124979504	2.7910289651138864
Cu	2.3828596631564878	0.2503663330611765	-2.0523615351586360
Cu	0.2841992212981204	1.3076681989250556	-1.6524262029430759
Cu	1.9033014308528333	1.2370047770138193	0.2027008766546281
Cu	0.8922952400008928	-0.8900737468445463	-0.5750991622241379
Cu	-0.5404836663367849	1.1180658513990682	0.6129554691473249
Cu	-0.9570672788952255	-1.4469832072155153	0.9020712835197084
Cu	-1.5567944575723871	-0.1867584464522558	-1.1707076275515806

Cu_{14}

14

Cu	-1.5064211197648643	-1.1504634927655051	-1.9738015919927001
Cu	0.2850445695919248	-1.4049953074800481	-0.2832990808854614
Cu	-1.7479401331172699	-0.2197423604151414	0.3390136609056000
Cu	0.8574372014476861	-1.1812723100312752	-2.5874903929594151
Cu	2.1495825722830002	-0.6587457106749230	1.2063889726418520
Cu	-0.1476853058457177	-0.8733742169661358	2.0121343390243052
Cu	-2.8119251937556307	0.8624134475877554	-1.5765026344506321
Cu	-1.7027171410248698	2.1364280990540347	0.1727988885108118
Cu	-1.1896051488582220	1.2790867690511636	2.4008808691286898
Cu	-0.3673148704725300	0.8693784675795868	-1.5264464248981486
Cu	0.5058964795984178	1.1628982374399204	0.7263829519730578
Cu	1.2138401700151640	0.8158769860743664	2.9961221759074661

Cu 1.9206242836922414 0.3293134526568302 -1.0186908241669439

Cu 2.5411836362106648 -1.9668020611106414 -0.8874909087384708

$Cu_{n-1}Pt$

Cu_3Pt

4

Cu 0.3191260591268019 -1.0335584513994913 -0.4878270528448070

Cu 1.9936341801562207 0.4578693102309703 0.2927525900352634

Cu -1.9833691271850284 -0.4845806761173801 -0.3110534508408591

Pt -0.3293911120979942 1.0602698172859011 0.5061279136504027

Cu_5Pt

6

Cu -1.3004724989168235 -0.2225663535725104 0.5297095880446090

Cu 2.5132612655813382 0.4103331721245755 -1.0480388440633241

Cu -1.5038113407831428 -2.0152828546637203 -0.9437713896625048

Cu 0.5067119222652678 -0.8407576643791881 -1.0300563056908434

Cu -1.0046414446767216 1.6080578246732991 1.9981500676061117

Pt 0.7889520965300818 1.0602158758175353 0.4940068837659517

Cu_7Pt

8

Cu 1.6402065900930136 -0.5401000287910911 0.4210409350864488

Cu -2.2289820475132833 -0.5409537096406591 0.3193665455764361

Cu -1.0057391955007784 1.1943434932370991 -0.8504993324750352

Cu -0.2892283300725591 -1.1175656543728500 -0.9264898036924869

Cu -0.1548060691915705 -1.9015520751170722 1.3124727923304356

Cu -0.3567355506401455 0.4676698568150943 1.3826737833250959

Cu 1.1988722668822067 0.4982039040062389 -1.8125086421722401

Pt 1.1964123359431031 1.9399542138632402 0.1539437220213458

Cu_9Pt

10

Cu -0.8404438273133099 -0.0011101312757162 -1.2826413646718571

Cu -1.4878413977132414 1.9946699320936752 -0.0175050087491826

Cu 0.8607734506282672 -1.3014195120632053 -0.0105609135316271

Cu 0.8570411542966898 1.3038542631851193 -0.0102552716666171

Cu -2.9524759870020354 -0.0041401188424537 -0.0291216748465146

Cu -1.4816952693854741 -1.9987838893866243 -0.0177003647896417

Cu 1.5238191569721398 0.0016659809725859 1.9863533805173539

Cu 1.4636285216116374 0.0022235630785374 -1.9653713852675772

Cu 2.9122393795718633 0.0040885992023920 -0.0029303199461985

Pt -0.8550451816665277 -0.0010486869643103 1.3497329229518620

$Cu_{11}Pt$

12

Cu -0.6613789694635059 -0.2502452084438680 -1.9843630467986539

Cu -0.6395431894388555 -0.2507963890330451 1.9916912256909178

Cu -2.9255124219143820 0.4854336980673555 -1.1992229900313038

Cu 1.9047343445500680 -0.9065172110519377 -2.1012264784373409

Cu -2.9117721605188391 0.4851547186317990 1.2307549307918269

Cu 1.1906565550004231 1.2299960858414494 1.1961453221607421

Cu -1.7783703681508403 -1.2256552289335207 0.0092941916086140

Cu 1.9277020320702096 -0.9069076718435980 2.0802068507648102

Cu 1.1776196343060068 1.2301025882297338 -1.2085037400978429

Cu 2.9895528185928857 -0.0504183004142735 -0.0162655814793240

Cu -0.9516583806597207 1.2232848633642206 0.0051872192274001

Pt 0.6779701056265441 -1.0634319444143205 -0.0036979033998676

Below, we provided the XYZ coordinates of the lowest energy H_2/Cu_n and $H_2/Cu_{n-1}Pt$ systems.

H_2Cu_n

H_2Cu_4

6

Cu 0.2308316908032833 1.6942161926017576 -1.0915854358724637

Cu -0.0286642058560300 -0.2124326914922712 0.1373940219835461

Cu 2.0873716027686644 0.8889806712745862 0.0835106808898445

Cu -1.8723187687251208 0.6656059084712851 -1.0954299567036667

H -0.6146428753501816 -1.5383112313224139 0.8659169835611493

H 0.1974225563593937 -1.4980588495329386 1.1001937061415923

H_2Cu_6

8

Cu -0.8988131583255203 -0.7899321865244762 -0.3874292078548276

Cu 2.7048049966196803 0.0555024153746739 -2.0432166071091915

Cu -1.3048024656379162 -2.3235374792230381 -2.1145464206157536

Cu 0.7205086010299162 -1.1666281574403836 -2.1354190214350481

Cu -0.4702132559970025 0.7384318066150080 1.3689469621468948

Cu 1.1351639850716566 0.3792993918369927 -0.3331722616430035

H -1.0768662440979409 1.8569811461108312 2.6076289557422889

H -0.8097824586628750 1.2498830632503815 3.0372076007686317

H_2Cu_8

10

Cu 0.8378974653006459 1.6948530980878438 0.6269626825082071

Cu 1.1249339531526275 -0.6766378283626366 0.9225738557842753

Cu -2.5055530745263948 -0.5663407734961297 1.2788236085225364

Cu -1.3028786110637753 0.9172621145951858 -0.1529498508773734

Cu -0.8232404885078832 -1.4386595381994773 -0.2636987050683413

Cu -0.5728364291359949 -1.8319294425472386 2.1376524003570871

Cu -0.5848142133706329 0.5696439836307086 2.1673140398645838

Cu 0.6691827511689219 0.1115282563902618 -1.3423947351558283

H 1.4451690935227450 0.2950902161872158 -2.8930556970831169

H 1.7121395534597426 0.9251899137142612 -2.4812275988520387

H_2Cu_{10}

12

Cu -0.3713980129703494 -0.5604067924273686 1.3028775032752211

Cu -0.3105520817354108 -0.8099234229900567 -1.1710420618741484

Cu -1.4865173929069684 1.0390121650530642 -0.1357489705891908

Cu 1.6663421025806180 -1.5103363412662691 0.1899041980943288

Cu 0.9752756724820415 0.9715262999760892 -0.0614804691608306

Cu -2.3838386968932257 -1.2424474093181033 0.0732122877685608

Cu -0.3912771627177563 -2.7246350939475503 0.2711661588596002

Cu 1.8652146708818513 0.0744367311695804 1.9730299074617843

Cu 1.9511693162554984 -0.2835201438140604 -1.8490298793013622

Cu 3.3617546861727750 0.2760079729799720 0.0590000619161888

H -2.7702762081153978 2.1532090898677385 -0.3064192964508425

H -2.1058968930336777 2.6170769447169491 -0.3454694399993219

H_2Cu_{12}

14

Cu -0.2468173307891455 -0.1165506203150350 -1.9813967121567639

Cu 1.1093449117413954 -0.3465015936519436 0.0465020999115957

Cu -0.3304562808918750 0.2220534098405267 1.9532546988544688

Cu -2.6064740728604203 0.2472598679072426 -1.3520466353850225

Cu 2.3067136281011162 -0.2188994878330490 -1.9989223470599846

Cu -2.6598107408586866 0.4597418613809161 1.1712718340411161

Cu 1.1553516414930876 2.0010103679788607 1.0479245305610245

Cu -1.2396222298012560 -1.1023867455460739 0.0653301495047760

Cu 2.2192099485445347 0.1255401499945759 2.0895035550885517

Cu 1.2063907620383993 1.8009153275742502 -1.3308243173489682

Cu 3.1345889136849650 0.9560173022960488 -0.0200049567677940

Cu -0.8733986912797693 1.3950994468093505 -0.1413914324678167

H -1.1995324937502776 -2.8017754860321107 0.1510067513927744

H -1.9754879653720572 -2.6215238004035619 0.2997927818320285

$H_2Cu_{n-1}Pt$

H_2Cu_3Pt

6

Cu 0.4976068561110001 -1.7704997917635055 -0.9069681578512157

Cu 2.0000041798262220 -0.3177476865903959 0.2350903956084398

Cu -1.7721254175200283 -1.2898969659827824 -0.8685033042186987

Pt -0.2488718363164413 0.4950199239681776 -0.0341093941802040

H 0.4811818078753767 1.8445855983574564 0.3414726895242168

H -0.9577955899761204 1.0385389220110532 1.2330177711174635

H_2Cu_5Pt

8

Cu -1.6092671549758926 -0.9213851829694182 0.0449012722155420

Cu 2.2451638182842188 0.1801651959565742 -1.0731482962037830

Cu -1.5078092453328686 -2.6650793673767224 -1.5419418481321596

Cu 0.3697793045208115 -1.2535793766060497 -1.3126799636433795

Cu -1.6885733750606811 0.8259409738416732 1.6482539648784584

Pt 0.3561153095380369 0.6347091826559055 0.3698893119141307

H 0.0848790972010089 1.7400856431169860 1.5181187254843813

H 1.7497122458253589 1.4591429313810522 0.3466068334867973

H_2Cu_7Pt

10

Cu 1.1045151907066160 -1.1634220949033267 0.2306866999738837

Cu -2.6600569907309657 -1.0221838372043521 0.0901602934691557

Cu -1.3491677514187383 0.7850617378034652 -0.9000470426366700

Cu -0.7628443951003394 -1.5437428270137179 -1.2265888825136635

Cu -0.7983449120065682 -2.5024795283969885 0.9489263465145417

Cu -0.8234338223194406 -0.1204485243593076 1.3257450627557379

Cu 0.8114753558130197 0.0416497201335115 -1.9719435624007620

Pt 0.8778202709652465 1.3515911261551423 0.1901873857108942

H 1.2020105630920863 2.4368319300583199 1.2770698678957064

H 2.3980264909990883 1.7371422977272655 0.0358038312311635

H_2Cu_9Pt

12

Cu -0.6274227425952041 -0.3577586034851432 -1.7166833946309126

Cu -1.1967250034406192 1.8436360809585040 -0.9112822783210586

Cu 0.9948761512453199 -1.4182227689116207 -0.2022291354432006

Cu 1.1075931773164047 1.0824861637879994 -0.7083497952425422

Cu -2.7960692913879193 -0.0098075041179690 -0.5201461579313271

Cu -1.3665181511144855 -1.9820079141266813 -0.0824600548933834

Cu 1.6565565777044675 0.1872406932218418 1.4836001066179136

Cu 1.7192122246393797 -0.5633552722529629 -2.3229039341796547

Cu 3.1579197451302576 -0.2397768245380583 -0.3120915260515904

Pt -0.8434019440378631 0.2352988390891468 0.9865371875035027

H 0.1308007498571389 0.4675097546409255 2.3050583988251070

H -1.9368214933168737 0.7547573557339984 2.0009505837471462

$H_2Cu_{11}Pt$

14

Cu -0.9315460000669482 0.3925665917412982 -2.5286014139560127

Cu -0.9056742150981041 0.3642696325861596 1.8429078845121829

Cu -3.0190155075451477 0.8469638249400173 -1.5285046398340381

Cu 0.8309382277210347 -1.0437249654631913 -1.6680198464589537

Cu -3.0299658116896309 0.7034630675613887 0.8461797564364915

Cu 1.0384079572168972 1.5192970651608366 0.9534055198480367

Cu -1.3956680753119759 -0.6770547613335864 -0.3931765984877096

Cu 2.6227374716092555 0.1278584636805906 2.0992688399128561

Cu 0.9673846917144591 1.3635099270883106 -1.4539139619241368

Cu 2.7364009928235014 0.1111623725731263 -0.3606313128928491

Cu -1.1156027098897816 1.7363252287907667 -0.3014366132568966

Pt 0.8843007610950000 -1.0748823208587446 0.8170173836063892

H 0.1543834729865363 -2.0240063529360839 1.8421844815727866

H 1.1629187444348972 -2.3457477735309311 -0.1666794790781481
